# Revealing neural correlates of behavior without behavioral measurements

**DOI:** 10.1038/s41467-019-12724-2

**Published:** 2019-10-18

**Authors:** Alon Rubin, Liron Sheintuch, Noa Brande-Eilat, Or Pinchasof, Yoav Rechavi, Nitzan Geva, Yaniv Ziv

**Affiliations:** Department of Neurobiology, Weizmann Institute of Science, Rehovot, 76100 Israel

**Keywords:** Neural decoding, Learning and memory, Neural circuits

## Abstract

Measuring neuronal tuning curves has been instrumental for many discoveries in neuroscience but requires a priori assumptions regarding the identity of the encoded variables. We applied unsupervised learning to large-scale neuronal recordings in behaving mice from circuits involved in spatial cognition and uncovered a highly-organized internal structure of ensemble activity patterns. This emergent structure allowed defining for each neuron an ‘internal tuning-curve’ that characterizes its activity relative to the network activity, rather than relative to any predefined external variable, revealing place-tuning and head-direction tuning without relying on measurements of place or head-direction. Similar investigation in prefrontal cortex revealed schematic representations of distances and actions, and exposed a previously unknown variable, the ‘trajectory-phase’. The internal structure was conserved across mice, allowing using one animal’s data to decode another animal’s behavior. Thus, the internal structure of neuronal activity itself enables reconstructing internal representations and discovering new behavioral variables hidden within a neural code.

## Abstract (web summary)

Neuronal tuning is typically measured in response to a priori defined behavioural variables of interest. Here, the authors use an unsupervised learning approach to recover neuronal tuning with respect to the recorded network activity and show that this can reveal the relevant behavioural variables.

## Introduction

Most neurons in the brain do not receive direct inputs from the external world; rather, their activity is governed by their interactions with other neurons within and across brain circuits. Despite this fact, studies in neuroscience typically focus on neuronal responsiveness to an examined external variable (i.e., a neuronal tuning curve). Rooted in the emergence in the 1950s of electrophysiological techniques for recording from single neurons in vivo, this ‘neural correlate’ approach has opened the door to studying how specific brain circuits form internal representations and has led to seminal breakthroughs. Examples of such breakthroughs include the discoveries of orientation tuning in the visual cortex^1^, hippocampal place cells^2^, and entorhinal grid cells^3,4^. However, while such analyses remain invaluable for many neuroscientific studies, they are limited to a priori defined external variables, overlooking other variables that were not measured or considered relevant^5,6^. With traditional electrophysiological techniques that permit recordings from only a small number of neurons, no feasible alternatives to the neural correlate approach existed. Recent advances in multi-electrode and optical imaging technologies enable simultaneous readout of activity from large neuronal populations, permitting a qualitatively different approach to study the neural code—via the attributes of neuronal activity itself.

Indeed, unsupervised learning has been shown to efficiently expose variables of interest in high-dimensional neural data^7–16^. However, whether such an approach could be used to expose unknown encoded variables and characterize differences between the computations undertaken by different brain regions has remained unclear. In principle, the relationships between neuronal population activity patterns give rise to a structure within the neuronal activity space that inherently reflects internal representations. Thus, the internal structure of neuronal activity should differ across brain circuits according to their distinct computational roles. Furthermore, characterizing the activity of single neurons with respect to this structure (rather than relative to any pre-selected external variable) could yield ‘internal tuning curves’, which capture the properties of classically computed tuning curves but are obtained without a priori knowledge or assumptions about the encoded variables. Thus, the realization of such an approach could facilitate the investigation of neural coding even in brain circuits for which little is known about the identity of the variables they encode, and circumvent biases associated with the naïve application of the neural correlate approach.

## Results

### Internal representation of space in the hippocampus

**Fig. 1:**
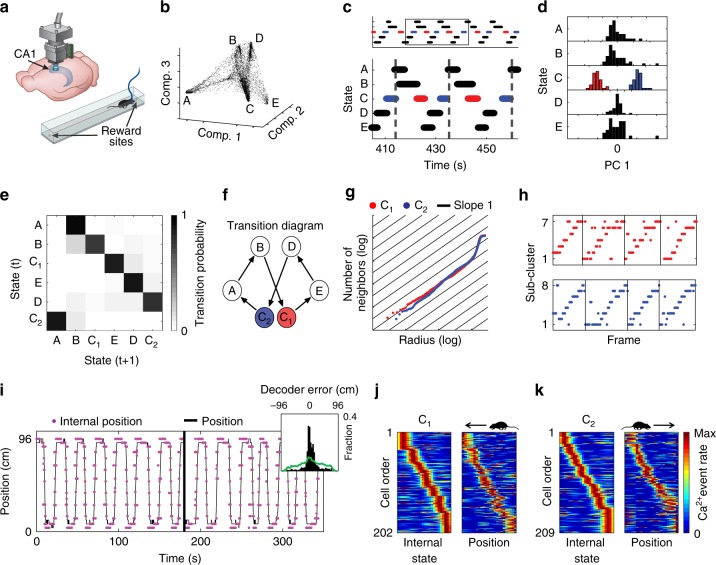
Hippocampal internal representation of position obtained without relying on behavioral data. **a** Schematic of Ca^2+^ imaging via a miniaturized head-mounted microscope. Mice ran back and forth on a linear track. **b** The distribution of data points in the reduced dimensional space of neuronal activity forms a small number of dense clusters (network states). Each data point corresponds to the neuronal activity within a single time frame. **c** The temporal structure of segmented data reveals a cyclic pattern of transitions between network states. **d** PCA applied separately to the segment-level activity patterns of each state indicated that only state C consists of two completely separate clusters (red and blue, subtypes C_1_ and C_2_, respectively). A histogram of the projections on the first principle component is presented for each state. **e** The transition matrix (i.e., the probability of a segment belonging to cluster *i* given that the preceding segment was in cluster *j*) shows a stereotypical pattern of transitions between network states. **f** Illustration of the structure of network states and the stereotypic pattern of transitions between them. **g** Estimation of the internal dimension calculated separately for data taken from segment subtypes C_1_ (red) and C_2_ (blue): Cumulative number of neighboring data points as a function of the radius threshold in the reduced dimensionality space. The slope of the data is close to one (black lines), indicating a dimension of one. **h** Sub-clusters within segments of subtype C_1_ (top) and C_2_ (bottom) exhibited a stereotypic temporal structure. Black vertical lines indicate concatenation points between segments within the same state subtype. **i** The reconstructed internal position (magenta) and the actual position of the mouse (black) on the linear track. Inset, distribution of the error in the reconstruction of position during running (black) versus shuffled data (green). **j**, **k** Internal tuning curves, relative to the network’s internal state (left), and the external tuning curves, relative to the position of the animal (right) for neurons active during C_1_ (**j**) and C_2_ (**k**). Neuronal activity within segment subtype is presented next to the activity of the same cells during leftward (**j**) or rightward (**k**) running

First, we sought to determine whether coding properties could be extracted from the internal structure of neuronal activity by studying a brain circuit known to encode a canonical variable. Thus, we focused on the dorsal CA1 of the hippocampus, a circuit in which many neurons are tuned to spatial position^2^. We used miniaturized head-mounted microscopes^17^ to image Ca^2+^ dynamics in GCaMP6-expressing hippocampal CA1 neurons in freely behaving mice. During imaging, mice ran back and forth along a linear track to collect water rewards on both ends of the track^18^ (Fig. 1a). After detecting cells and extracting their Ca^2+^ events from the imaging data^18,19^, we constructed neuronal ensemble activity vectors of instantaneous neuronal activity sampled at fixed time bins. To explore the relationships between the ensemble activity patterns, we sought to embed the data in a reduced dimensional space of neuronal activity. Non-linear dimensionality reduction techniques enable such embedding even if the data lays on a non-linear manifold, making them, in many cases, more suitable than linear methods^20^ (Supplementary Figs. 1 and 2). Applying the non-linear dimensionality reduction algorithm Laplacian Eigenmaps^21^ (LEM) to the activity vectors (Fig. 1b and Supplementary Movie 1) revealed a structure in the reduced dimensional neuronal activity space, with a high density of data points within a small number of clusters (clusters A, B, C, D, and E in Fig. 1b, Supplementary Figs. 3a, 4 and 5a), suggesting that throughout the imaging session, the network leaped between discrete states. Note that applying principal components analysis (PCA), the commonly used linear dimensionality reduction method, did not reveal discrete network states in the CA1 activity (Supplementary Fig. 1a). Temporally segmenting the data points into time intervals during which the network remained within a given state, revealed a recurring cyclic pattern of transitions between network states (Fig. 1c). Within a cycle, each state appeared only once, except for state C, which appeared twice, in two different phases. Further analysis revealed that the set of temporal segments of state C consisted of two separate state subtypes (C_1_ and C_2_; Fig. 1d), each corresponding to one of the two different phases (red and blue segments in Fig. 1c). These analyses allowed us to portray the intricate structure of network states and the pattern of transitions between them (Fig. 1e, f).

By sorting the behavioral data according to the specific states of the network, we found that the different network states corresponded to different behaviors and locations along the linear track (Supplementary Movie 2). Specifically, states A and E, which were symmetrically located within the internal structure (Fig. 1b), corresponded to drinking at the left and right sides of the track, respectively. Similarly, the symmetric states B and D corresponded to turning at the two sides of the track. Consistent with this, we found that individual neurons were highly tuned to either drinking or turning at one end of the track (Supplementary Fig. 6). Note that such a result would have been missed by a conventional analysis of spatial tuning that ignores information about non-spatial external variables. States C_1_ and C_2_ corresponded to epochs of running in different directions along the linear track, consistent with the characteristics expected for spatial coding in one-dimensional environments^22,23^. Thus, the internal structure of neuronal activity exposed discrete sets of network states that corresponded to different combinations of locations and behaviors, without needing to a priori hypothesize that these specific behavioral states are encoded by hippocampal neurons.

We next attempted to use the structure of the neuronal activity to characterize the internal representation of space at a finer resolution, focusing our analysis on states C_1_ and C_2_. We applied the dimensionality reduction procedure separately for the data within C_1_ and C_2_. Then, we estimated the internal dimension of the data^24^ (Supplementary Fig. 7) and the topology for each state subtype, and found that both C_1_ and C_2_ were one-dimensional (Fig. 1g), with one component, no holes, and no spaces, consistent with the topology of a line. Following sub-clustering the data points within C_1_ and C_2_, we found an ordered pattern of transitions between the sub-clusters throughout each segment, which monotonically covered a one-dimensional continuum of network states (Fig. 1h, Supplementary Figs. 5b, c and 8a). Consistent with these observations, the trajectory of the network within the internal structure of neuronal activity reflected the trajectory of the mouse along the track, permitting accurate reconstruction of position (Fig. 1i; permutation test, *p* < 0.001 for each mouse, *N* = 4). Note that because the reconstructed trajectory is not calibrated to the internal symmetry of the encoded variable or to its identity, we set the reflection degree of freedom of the internal representation to match the position of the mouse. We then calculated the internal tuning curves of individual neurons, i.e., the activity of single neurons with respect to the different network states. The internal tuning curves had similar properties to those of their corresponding external tuning curves (i.e., place fields; Fig. 1j, k, Supplementary Figs. 9a, 10a; permutation test, *p* < 0.001 for each mouse, *N* = 4). Overall, these results demonstrate that the relationships among neuronal activity patterns themselves are sufficient to reconstruct the hippocampal representation of space.

### Internal representations in the medial prefrontal cortex

**Fig. 2:**
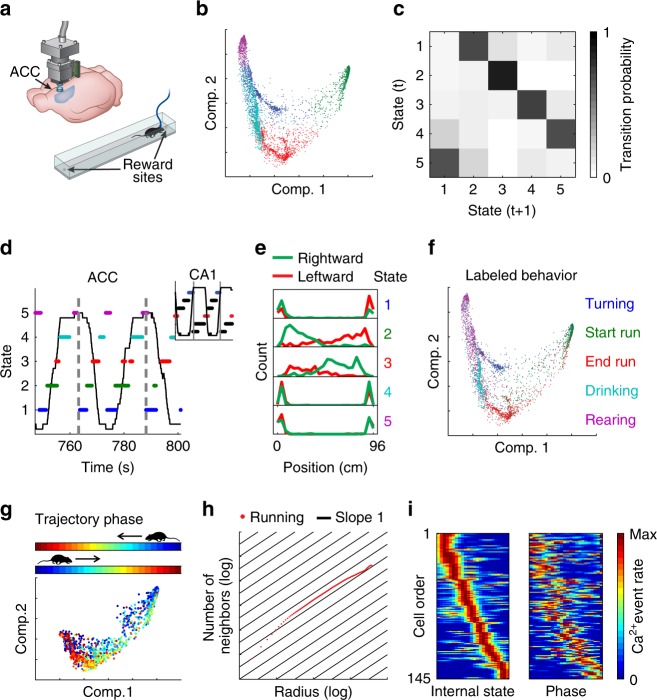
ACC activity reveals a schematic representation of behavior along the linear track. **a** Schematic of Ca^2+^ imaging via a miniaturized head-mounted microscope in the ACC of a freely behaving mouse. Mice ran back and forth to collect rewards at the two ends of a linear track. **b** The distribution of data points in the reduced dimensional space of neuronal activity. Each data point corresponds to the neuronal activity within a single time frame. The data are clustered and colored to indicate each of the different network states. **c** The segment-level transition matrix shows a stereotypical pattern of transitions between the different network states. **d** A stereotypic temporal structure of network states (marked by different colors) is observed during exploration of the linear track (animal position is shown in black). A full running cycle (bounded between vertical gray lines) corresponds to two cycles of transitions between network states. Inset, hippocampal data in which a full running cycle (bounded between vertical gray lines) corresponds to a single cycle of transitions between network states. **e** The distribution of mouse position along the linear track is symmetrical for each of the network states. **f** The same distribution presented in (**a**) with data points colored according to behavioral labeling. **g** Distribution of data points in the reduced dimensional space of neuronal activity for the two clusters corresponding to running. Data points are colored according to trajectory phase, i.e., the distance of the mouse from the start of the track (opposite end for each running direction). **h** Estimation of internal dimension for the data points in (**g**) (running states). Cumulative number of neighboring data points as a function of the radius threshold in the reduced dimensionality space, plotted on a log–log scale. The slope of the data is close to one (black lines), indicating a dimension of one. **i** For each neuron, we calculated its internal tuning curve, relative to the internal state of the network (left), and the external tuning curve, relative to the trajectory phase of the animal (right)

Our results in the previous section demonstrated how our analysis can reveal internal representations in the hippocampus, a brain region in which the encoding of a known variable (place) is dominant. We next examined whether a similar analysis can reveal internal representations using recordings from brain circuits that have not been associated with a canonical encoded variable. To this end, we focused on the anterior cingulate cortex (ACC), a sub-region of the medial prefrontal cortex (mPFC) that is involved in multiple high-order cognitive processes^25–28^. We conducted Ca^2+^ imaging in the ACC of freely behaving mice that performed the same linear track exploration task described above for hippocampal imaging (Fig. 2a). Dimensionality reduction of the neuronal activity revealed a high density of data points within a small number of clusters (Fig. 2b and Supplementary Figs. 3b and 4), similar to our observations in the hippocampus. Next, we clustered the data points into different network states and calculated their transition matrix (Fig. 2c), which exhibited a recurring cyclic pattern (Fig. 2c, d). In contrast to our observations from the hippocampal data, in the ACC, the representation of behaviors on both sides of the track converged onto the same network states (Fig. 2d). Consistent with this observation, the distribution of the animal’s position along the linear track was symmetrical for each of the identified network states (Fig. 2e). Furthermore, by sorting the behavioral data according to the network states, we found that each state corresponded to a distinct behavior, irrespective of the position of the mouse. We termed these behaviors ‘Rearing’, ‘Turning’, ‘Start run’, ‘End run’, and ‘Drinking’ (Supplementary Movie 3). We confirmed this clustering-based classification using manual labeling of the mouse’s behavior (Fig. 2f).

To study the structure of the neuronal activity in the ACC at a finer resolution, we focused on the network states linked to running (‘Start run’ and ‘End run’). We found that neuronal activity represented the position of the mouse relative to the start and end points of each track traversal, regardless of the running direction (i.e., the trajectory phase; Fig. 2g and Supplementary Fig. 11). The internal dimension of the data during running states was one (Fig. 2h), with one component, no holes, and no spaces, suggesting a topology of a line. Consistent with these observations, constructing internal tuning curves revealed individual neurons that were tuned to a specific trajectory phase, namely, ‘trajectory-phase cells’ (Fig. 2i and Supplementary Fig. 12a, b). The internal tuning captured the external tuning of the same neurons to the trajectory phase (Fig. 2i and Supplementary Fig. 10b; permutation test, *p* < 0.001 for each mouse, *N* = 3). The activity profile of neurons in the ACC could not be explained by the animal’s speed or acceleration per se, since the same value of either of them will be met twice within a single-direction trajectory, whereas our data shows that ACC neurons tended to fire only in one phase within such a trajectory (Supplementary Fig. 13). Furthermore, neuronal activity in the ACC is more accurately explained by trajectory phase than by time or speed and acceleration (explained variance = 0.67, 0.48, and 0.40 for trajectory phase, time, and speed-by-acceleration, respectively; Supplementary Fig. 14). Together, these results demonstrate that even in a brain circuit in which less is known about the identity of the encoded variables, our analysis can expose key properties of the internal representation, including the encoding of a previously unknown variable.

### Different internal representations in the ACC and the CA1

**Fig. 3:**
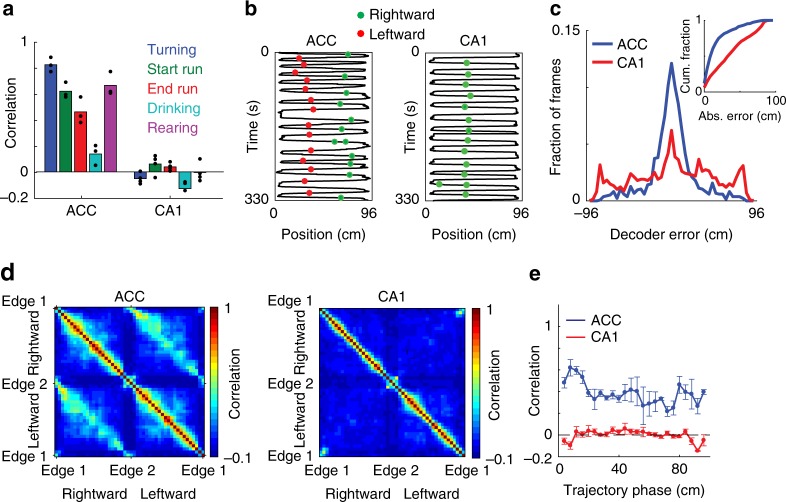
The different internal structures in the ACC and the hippocampus reflect different representations of locations and actions. **a** Pearson correlation between ensemble activity patterns from the two sides of the linear track, for each behavioral state, for data recorded in the ACC (left) and hippocampal CA1 (right). Ensemble activity patterns consist of concatenated epochs from a given behavioral state on the same side of the track. Data show means for *N* = 3 mice in the ACC and *N* = 4 mice in the CA1. **b** An example trajectory-phase cell recorded in the ACC (left) and an example place cell recorded in the hippocampus (right). The black lines show the positions of the animals, and the green and red dots show the activity of the neurons during rightward and leftward running, respectively. **c** Distribution of decoding error of trajectory phase for data recorded in the ACC (blue) and CA1 (red). The decoder was trained on data from running in one direction and tested on data from running in the other direction. Inset, cumulative fraction of the absolute decoder error. **d** Pearson correlation between ensemble activity patterns across different spatial locations on the linear track, for data recorded in the ACC (left) and CA1 (right). Ensemble activity patterns consist of concatenated epochs from a given location, separated according to the two running directions. **e** Pearson correlation (mean ± SEM) between ensemble activity patterns from the two sides of the linear track, given the same trajectory phase, for data recorded in the ACC (blue) and CA1 (red). Ensemble activity patterns are defined as the mean event rate for each neuron given a spatial bin and running direction. Data in **c**-**e** pooled from N = 3 mice in the ACC and *N* = 4 mice in the CA1

Having found different structures of neuronal activity in the ACC and hippocampus under the same behavioral task, we sought to further characterize the differences between their internal representations of locations and actions. Neuronal activity in the ACC, but not in the hippocampus, was similar during epochs of the same behavioral state at opposite sides of the track (Fig. 3a and Supplementary Fig. 15; one sample *t-*test, *p* < 0.05 and *p* > 0.05 for each behavioral state for ACC and CA1, respectively). The distinction between the neuronal coding in the hippocampus and ACC was also evident when examining the tuning of individual cells (Fig. 3b and Supplementary Fig. 12). To further substantiate the differences between the encoding of trajectory phase and position, we devised a phase decoder, which allowed us to accurately infer the trajectory phase in a given direction in the ACC, when the decoder was trained on the relationship between neuronal activity and the trajectory phase in the opposite direction (blue curve in Fig. 3c; permutation test, *p* < 0.001 for each mouse, *N* = 3). Notably, we could not predict the trajectory phase when applying the same analysis to hippocampal data (red curve in Fig. 3c; permutation test, *p* > 0.05 for each mouse, *N* = 4). Consistent with these findings, population activity in the ACC was correlated between symmetrical spatial locations for the opposite running directions along the linear track (Fig. 3d, e). In contrast, in the hippocampus, neuronal representations differed considerably between the two sides of the track (Fig. 3d, e), and were more sharply tuned to position than in the ACC (Supplementary Fig. 16). Overall, we found that ACC neurons are spatially tuned, but the nature of their spatial representation is markedly different from the classical spatial tuning properties of hippocampal neurons.

### Representation of head direction during wake and REM sleep

**Fig. 4:**
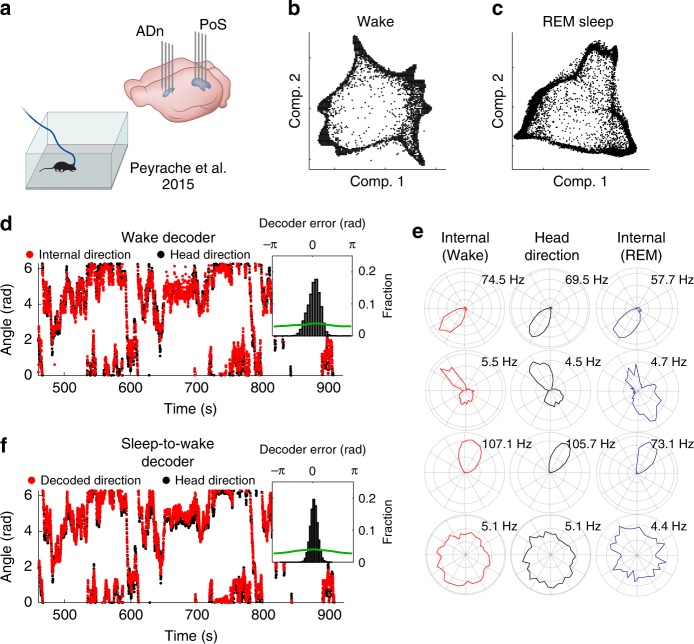
Internal representation of head direction during wake and REM-sleep periods. **a** Data were obtained from dense electrophysiological recordings from the ADn and PoS of mice foraging for food in open environments^29^. **b**,**c** The distribution of data points in the reduced dimensional space of neuronal activity exposes a ring topology during wake periods (**b**) and during periods of REM sleep (**c**). Each data point corresponds to the neuronal activity within a single time frame. **d** The reconstructed internal direction (red) and the actual head direction (black). Inset, distribution of the error in the reconstruction of head direction (black) versus shuffled data (green). **e** The internal tuning curve, relative to the state of the network (left, red), the external head-direction tuning curve, relative to mouse behavior (center, black), and the internal tuning curves during periods of REM sleep (right, blue) for four representative cells. **f** The decoded head direction (red) and the actual head direction (black). Inset, distribution of the decoding error (black) versus shuffled data (green). The decoder is based on internal tuning curves obtained exclusively from sleep

Since our approach does not rely on behavioral measurements, we next asked if we can use it to expose internal representations even when there is no correspondence between neuronal activity and the external stimulus or behavior. It has been shown that pairwise correlations between head-direction neurons in the anterior dorsal nucleus of the thalamus (ADn) and the postsubiculum (PoS) are preserved during sleep^29^. Therefore, we analyzed published electrophysiological recordings from the ADn and PoS^29,30^ of mice foraging for food in open environments (Fig. 4a), and sought to compare the internal structure of neuronal activity during wake and sleep periods. We constructed neuronal ensemble activity vectors, as described above for the Ca^2+^ imaging data, and applied dimensionality reduction to the activity vectors, separately for wake and REM-sleep periods. This analysis exposed a ring structure in the reduced dimensional space of neuronal activity for both wake and REM data (Fig. 4b, c, Supplementary Figs. 1c, d, 3c and 8c), consistent with previous reports^31,32^. By quantifying the internal dimension^24^ and topology^13,33–35^ of the data (Supplementary Fig. 18a–d), we found a one-dimensional structure (Supplementary Figs. 17h and 19h), with one component, one hole, and no spaces (Supplementary Figs. 18e and 19i), providing an additional indication that the encoded variable is indeed characterized by a ring topology during both wake and REM-sleep periods. These properties are consistent with the encoding of head direction previously observed in the ADn^36^ and PoS^29,36,37^.

We used the temporal contiguity of the network states (Supplementary Fig. 17a, b) to reconstruct the trajectory of the network within the neuronal activity space and validated it against the head direction of the mouse. This analysis confirmed that the reconstructed trajectory reflected the actual head direction, up to the internal symmetries of a ring to reflection and rotation (Fig. 4d, Supplementary Fig. 17c, d, and Supplementary Movie 4). By accounting for the activity of each neuron relative to the network activity states of the entire recorded population, we calculated the internal tuning curves, and found them to be similar to the external tuning curves (Fig. 4e, Supplementary Figs. 17e–g and 9b). A similar analysis of the data recorded during REM sleep revealed that the internal tuning curves obtained exclusively during REM matched the head-direction tuning curves during wake periods (Fig. 4e, Supplementary Fig. 19c–g, and Supplementary Movie 5). Remarkably, based on the internal tuning curves obtained during REM sleep, we were able to accurately decode the head direction (up to the ring’s internal symmetry) while the mouse was awake and freely behaving (Fig. 4f). We also found that the data points in the reduced dimensional space covered the same set of internal network states during wake and REM-sleep periods (Supplementary Fig. 18j, k). These observations suggest that in certain brain circuits the internal structure of neuronal activity is conserved irrespective of the animal’s behavior.

### Conservation of the internal structure of neuronal activity

**Fig. 5:**
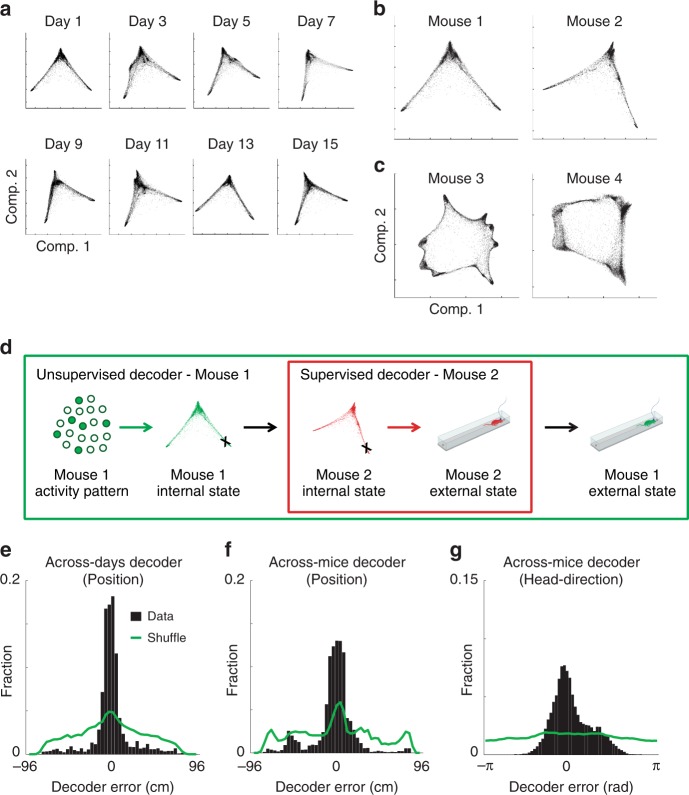
The internal structure of neuronal activity is maintained across days and across mice. **a** The distribution of data points in the reduced dimensional space of neuronal activity for hippocampal data from eight different days of the experiment. Note that the distribution of data points maintains a similar structure across days. **b**, **c** The distribution of points in the reduced dimensional space of neuronal activity for hippocampal data (**b**) and ADn/PoS data (**c**) from two different mice. **d** Workflow for the across-mice decoder. Neuronal activity in mouse 1 is associated with a specific network state within its internal structure of neuronal activity. Based on the similarity between internal structures, an analogous network state is found in mouse 2. This network state of mouse 2 is associated with a known external state. The decoded external state of mouse 1 is set as the same associated external state of mouse 2. **e**–**g** Distribution of decoding error for the across-days decoder for hippocampal data (**e**), the across-mice decoder for hippocampal data (**f**), and the across-mice decoder for ADn/PoS data (**g**)

If the internal structure of neuronal activity reflects the computational processes undertaken by the network, this structure should be an invariant property. To test this, we imaged neuronal activity in the hippocampus of mice that visited the same linear track on multiple days and found that the structure of neuronal activity remained similar over time (Fig. 5a and Supplementary Fig. 20), despite the substantial turnover of the active cells^18,38^ (41–64% overlap across days). The internal structure of neuronal activity in the hippocampus (Fig. 5b) and in the ADn and PoS (Fig. 5c) was also similar across different mice.

Based on the similarity between the internal structures across mice, we devised an across-mice decoder (Fig. 5d). First, we identified analogous activity patterns across data sets from different mice. Then, we inferred the behavior of mouse 1 using the mapping between the behavior and activity patterns in mouse 2. Specifically, we set the decoded external state (position) of mouse 1 as the same associated external state in mouse 2. We also used the same procedure to devise an across-days decoder. Using these decoders, we could accurately infer the position (Fig. 5e, f and Supplementary Movie 6) or head direction (Fig. 5g) of a mouse based on the mapping between the behavior and activity patterns in another mouse or day. Overall, these results demonstrate the conservation of internal structures of neuronal activity over time and across mice.

## Discussion

**Fig. 6:**
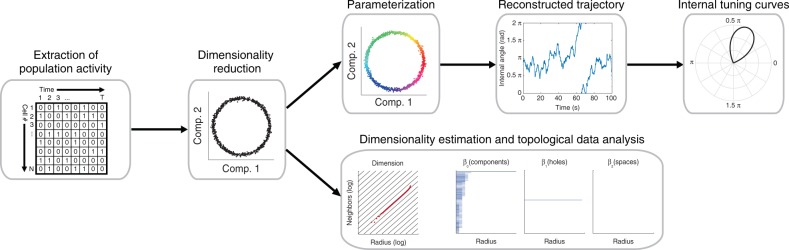
Key steps for unsupervised exposure of the internal structure of neuronal activity and the reconstruction of internal representations. First, the population neuronal activity is binarized and temporally binned into *N*-dimensional activity vectors (*N* = number of neurons). A dimensionality reduction algorithm is then applied to the activity vectors, embedding the data and exposing its internal structure within a low-dimensional space. Next, the activity in the reduced space is parameterized using a clustering algorithm. This allows the reconstruction of the trajectory of neuronal activity and the internal tuning curves of individual neurons as a function of the parameterized variable. In parallel, the dimension and topology of the data in the reduced space can be estimated to reveal key properties of the internal representations

Here, we introduce a new approach for studying the neural code based solely on the attributes of neuronal activity. By applying dimensionality reduction to large-scale neuronal data, we show that the internal structure of neuronal activity can reveal key properties of the neural code in a given brain circuit (Fig. 6). Previous studies have used dimensionality reduction methods to explore population-level coding of variables of interest in high-dimensional neural data^5,7,8,11,39–42^. Unlike previous applications of dimensionality reduction in neuroscience, we show that internal representations and tuning curves can be reconstructed from the internal structure of neuronal activity. Importantly, we demonstrate that such reconstructions can be used to expose internal representations even in brain circuits where the encoded variable is unknown. Furthermore, we demonstrate the conservation of the internal structure of neuronal activity over time and across mice, suggesting that the internal structure of neuronal activity is an invariant property of the network.

While in this work we used LEM for dimensionality reduction, it is likely that the approach we introduce here could accommodate other dimensionality reduction methods as well. Our analysis suggests, however, that non-linear dimensionality reduction methods are more suitable for studying the internal structure of neuronal activity, as they allow the extraction of a structure that accurately reflects internal representations in cases where linear methods fail^20^ (Supplementary Figs. 1 and 2).

Since our approach relies on minimal prior assumptions and is applied irrespective of the specific identity of the encoded variables, it could circumvent biases and limitations inherent to the standard method of calculating neuronal tuning curves. Furthermore, calculating internal tuning curves could alleviate the need to set non-adaptive and arbitrary boundaries between different behavioral states when calculating (external) tuning curves. For example, place fields are typically calculated after applying position and velocity thresholds to define periods of locomotion^38,43^, and only neuronal activity during those periods is considered in the analysis. Our results demonstrate that neuronal activity itself can be used to identify behavioral states and the boundaries between them, enabling an analysis that is derived explicitly from the neuronal activity data. Using our approach, we found a set of hippocampal states that corresponded to different behaviors. This included the identification of representations of two different actions that occur in the same place, a result that would have been missed by a conventional analysis of spatial tuning. Furthermore, we were able to zoom-in on the internal structure of the running states and use it to accurately reconstruct the position of the mouse along the track. Our analysis (Supplementary Figs. 2 and 8) implies that a denser recording (more neurons or higher temporal resolution)^44–46^ might enable such a reconstruction also during other behavioral states (e.g., turning and drinking).

Our approach allowed us to compare the internal representations in the hippocampus and the ACC while mice were performing the same behavioral task. Although both brain circuits exhibited a discrete set of network states that corresponded to different locations and behaviors, their internal structures of neuronal activity differed, reflecting different types of internal representations. The hippocampus represented a combination of locations and actions, and these representations differed between opposite sides of the track. In contrast, our analysis of recordings from the ACC exposed schema-like representations of distances and actions that are similar across the opposite sides of the track, including the encoding of a previously unknown variable—the trajectory phase. Previous work demonstrated that the mPFC is important for the assimilation of newly acquired information against prior knowledge, suggesting an underlying schematic organization of information^47^. Our findings are consistent with this notion, and specify how such schemas can be realized at the neural code level.

One major factor that governs the dimensionality of the observed neuronal dynamics is the complexity of the behavioral task^48^. In this study, we used a relatively simple behavioral task, i.e., repeated exploration of a linear track, yet we were able to expose different internal structures for the same task/behavior in the CA1 and ACC. This demonstrates that the complexity of the task was sufficient to expose coding properties that are specific to the studied brain regions.

One case in which the complexity of the behavioral task does not seem to affect the dimensionality of the neuronal dynamics is the head-direction system, where two tasks as different as explorative behavior and sleep exhibit a similar repertoire of neuronal activity patterns. A common practice is to train a decoder based on neuronal activity patterns defined during awake periods to identify similar ‘virtual trajectories’ during sleep^29,49,50^. Such analyses allow showing that certain patterns observed during wake periods occur at a higher probability than mere chance during sleep periods. In contrast to this approach, we used activity patterns defined during sleep to decipher the behavior of awake animals, which allows decoding performance to be validated against an actual observed behavior. Thus, the internal structure of neuronal activity can reveal internal representations even during sleep—when the correspondence between neuronal activity and the external stimulus or behavior is irrelevant. While it could be that in certain cases, no external variable can be associated with the exposed variable, our analysis of head-direction data during REM sleep demonstrates that even if we cannot validate the identity of the encoded variable, we can still use the internal structure, dynamics within it, and typical time constants to study network-level computations.

Estimations of internal structure, such as estimation of the intrinsic dimension and topological data analysis, can reveal the properties of the set of stimuli that were experienced by the animal (for example, the number of holes or areas within the arena that the animal cannot cross)^13,14,35^. Note, however, that while such properties reflect behavior or stimulus statistics, they are not necessarily inherent to the studied circuits. In contrast, there are cases in which the topology of the data cannot be explained by stimulus statistics. For example, even when there is no overt structure to the animal’s behavior or to the stimulus presented to it (e.g., during free exploration or during sleep), the neuronal activity within the animal’s ADn/PoS exhibits a ring topology^31,32^ because this circuit encodes the head direction of the animal (Supplementary Figs. 18 and 19). Thus, in addition to revealing features of the behavior or stimulus statistics, studying the internal structure of neuronal activity may also reveal coding properties that are inherent to the brain region of interest.

Recent studies have shown that different visits to the same familiar environment are encoded by different subsets of hippocampal neurons^18,38^. Consequently, it has been hard to reconcile how memories can be stably stored over the long term in a circuit that yields an ever-changing neural code. Our findings that the internal structure of neuronal activity remains stable over time raise the possibility that long-term memory stability is achieved, in part, through a stable relationship between neuronal population activity patterns. Such a stable relationship may also support the encoding of new information via its integration into a pre-existing structured code.

We also demonstrated the conservation of the internal structures of neuronal activity between mice. This conservation allowed us to infer the position or head direction of a mouse based on the mapping between behavior and activity patterns of another mouse. Thus, the analogy between internal structures allows the meaning of network states to be exported from one mouse to another (up to the symmetry of the internal structure). Note that the ability to decode behavioral states across individuals requires 2nd-order isomorphism^51^ (i.e., that the representation is not idiosyncratic). As task complexity increases, so does the number of alternative possible representations that different individuals may utilize. If different individuals apply different task strategies, which are associated with different internal structures of neuronal activity, then across individuals decoding might not be possible.

The internal structure of neuronal activity highlights general features of the computational task executed by a brain circuit. Consistent with this idea, recent studies have shown that the same neural circuit may similarly encode different external variables in different behavioral tasks^52–54^. For instance, non-spatial representations in the entorhinal cortex form a hexagonal grid-like pattern^53,54^, similar to the known spatial tuning of grid cells^3,4,55^. Since analogous tuning properties were observed in both cases, it is predicted that they would have the same internal structure of neuronal activity—a torus^56,57^. Thus, the internal structure of neuronal activity can serve as a fingerprint of the computations carried out by different neural circuits, enabling to investigate their function and even redefine their identity.

## Methods

### Animals and surgical procedures

All procedures were approved by the Weizmann Institute IACUC. We used male mice that were 8–12 weeks old at the beginning of the study. Mice designated for calcium imaging in the hippocampal CA1 were housed with 1–4 cage-mates, while mice used for imaging in the ACC were single housed. All cages contained running wheels. All surgical procedures were conducted under isoflurane anesthesia (1.5–2% volume). For hippocampal imaging, we used C57BL/6 wild-type mice, which underwent two surgical procedures: virus injection and glass tube implantation. We injected 400 nl of the viral vector AAV2/5-CaMKIIa-GCaMP6f^58^ (~2 × 10^13^ particles per ml, packed by University of North Carolina Vector Core) into the CA1 at stereotactic coordinates: −1.9 mm anterio-posterior, −1.4 mm mediolateral, −1.6 mm dorsoventral relative to the Bregma. The injected mice were allowed to recover in their home cage for at least 1 week before the subsequent surgical procedure. We next implanted a glass guide tube directly above the CA1, as previously described^18,38^. For ACC imaging, we used CaMKII-tTA and TRE-GCaMP6s double-transgenic mice (Jackson stock no. 003010 & 024742; referred to as CaMKII-GCaMP6), bred on a C57BL/6 background. They were implanted directly with a micro-prism lens (800 μm diameter) in the ACC. Stereotactic coordinates of the implantation were 1 mm anterior-posterior, 0 mm mediolateral (measured relative to the medial side of the prism), and −1.8 mm dorsoventral from Bregma.

### Preparatory process

For time-lapse imaging in freely behaving mice using an integrated miniature fluorescence microscope (nVistaHD, Inscopix), we followed a previously established protocol^18,38^. Briefly, at least 3 weeks after the surgical implantation procedure, we examined the expression of a Ca^2+^ indicator and tissue health by imaging mice under isoflurane anesthesia using a two-photon microscope (Ultima IV, Bruker, Germany) equipped with a tunable Ti:Sapphire laser (Insight, Spectra Physics, Santa Clara, CA). For the CA1-implanted mice, we inserted into the guide tube a ‘microendoscope’ consisting of a single gradient refractive index lens (0.44 pitch length, 0.47 NA, GRINtech GmbH, Germany). We selected for further imaging only those mice that exhibited homogenous GCaMP6 expression and appeared to have healthy tissue. For the selected CA1-implanted mice, we affixed the microendoscope within the guide tube using an ultraviolet-curing adhesive (Norland, NOA81, Edmund Optics, Barrington, NJ). Next, we attached the microscope’s base plate to the dental acrylic cap using light cured acrylic (Flow-It ALC, Pentron, Orange, CA). All mice were returned to their home cages for several days following the aforementioned procedure.

### Ca^2+^ imaging in freely behaving mice

We trained the mice to run back and forth on a 96-cm-long, 5-cm-wide linear track elevated 33 cm above the floor^18^. We placed the track in a square enclosure (160 × 160 cm) within the recording room. The walls of this 190-cm-high enclosure were curtains with visual cues of different colors and patterns (distal cues). Distinct visual cues were also scattered along the walls (4 cm high) of the linear track (proximal cues). To record mouse behavior, we used an overhead camera (DFK 33G445, The Imaging Source, Germany), which was synchronized with the integrated microscope. Both pre-training and imaging sessions consisted of five to seven 3-min-long trials, with an inter-trial interval of 3 min Ca^2+^ imaging was performed at 20 or 10 Hz in CA1 or ACC, respectively. The number of pre-training days and the behavior of the mice on the track were similar for the presented analyses (six pre-training sessions for recordings from the ACC and eight for recordings from the CA1; the number of track traversals was 68 ± 7 for CA1 and 64 ± 9 for ACC).

### Ca^2+^ imaging data processing

We processed the imaging data using commercial software (Mosaic, version 1.1.1b, Inscopix) and custom MATLAB routines as previously described^18,38^. To increase computation speed, we spatially down-sampled the data by a factor of 2 in each dimension (final pixel size of 2.3 × 2.3 μm). To correct for non-uniform illumination both in space and time, we normalized the images by dividing each pixel by the corresponding value from a smoothed image. The smoothed image was obtained by applying a Gaussian filter with a radius of 100 μm to the movies. Normalization also enhanced the appearance of the blood vessels, which were later used as stationary fiducial markers for image registration. We employed a rigid-body registration to correct for lateral displacements of the brain. This procedure was performed on a high-contrast sub-region of the normalized movies in which the blood vessels were most prominent. The movies were transformed to relative changes in fluorescence:

1 $$\frac{{\Delta F\left( t \right)}}{{F_0}} = (F\left( t \right) - F_0)/F_0$$

where *F*_0_ is the value for each pixel averaged over time. For cell detection, the movies were down-sampled in time by a factor of 5. We detected spatial footprints corresponding to individual cells using an established cell-detection algorithm that applies principal and independent component analyses (PCA and ICA, respectively). For each spatial footprint, we used a threshold of 50% of the footprint’s maximum intensity. Each pixel that did not cross the threshold was set to zero. After the cells were detected, cell sorting was performed to identify the spatial footprints that follow a typical cellular structure. This was done by measuring the footprint area and circularity and discarding those whose radius was smaller than 5 μm or larger than 15 μm or which had a circularity smaller than 0.8. In some cases, the output of the PCA/ICA algorithm included more than one component that corresponded to a single cell. To eliminate such occurrences, we examined all cell pairs with centroid distances < 18 μm; whenever their traces had a correlation > 0.9, the cell with the lower average event peak amplitude was discarded. The number of cells per mouse were 452, 497, 453, and 547 in the CA1 and 602, 521, and 603 in the ACC. To identify the same neurons across multiple imaging sessions, we used a probabilistic method for cell registration^59^, which estimates the probability of correct registration for each cell in the data set and the overall rates of registration errors.

### Detection of Ca^2+^ events

Ca^2+^ activity was extracted by applying the thresholded spatial footprints to movies at full temporal resolution (20 Hz) $\Delta F(t)/F_0$. Baseline fluctuations were removed by subtracting the median trace (20 s sliding window). The Ca^2+^ traces were smoothed with a low-pass filter with a cutoff frequency of 2 Hz. Ca^2+^ candidate events were detected whenever the amplitude crossed a threshold of 4 or 5 median absolute deviations (MAD) for GCaMP6s or GCaMP6f, respectively. We considered for further analysis only candidate Ca^2+^ events with an indicator decay time for GCaMP6s or GCaMP6f equal to or longer than 600 or 200 ms, respectively. In order to avoid the detection of several peaks corresponding to a single Ca^2+^ event, only peaks that were 4 or 5 MAD higher than the previous peak (within the same candidate event) and 2 or 2.5 MAD higher than the next peak, for GCaMP6s or GCaMP6f, respectively, were regarded as true events. We set the Ca^2+^ event occurrence to the time of the peak fluorescence. To mitigate the effects of crosstalk (i.e., spillover of Ca^2+^ fluorescence from neighboring cells), we adopted a conservative approach, allowing only one cell from a group of neighbors (pairs of cells with centroid distances < 18 μm) to register a Ca^2+^ event in any 200 ms time window. If multiple Ca^2+^ events occurred within ~200 ms in neighboring cells, we retained only the event with the highest peak $\Delta F/F_0$ value. If two neighboring cells had a correlation > 0.9 in their events, the cell with the lower average peak amplitude was discarded. After the events were identified, further event sorting was performed to find the cells with sufficient signal-to-noise ratios. This was done by measuring the event rate and the average event peak amplitude for each cell and discarding those whose event rate was smaller than 0.01 Hz or which had an average event amplitude smaller than 1% $\left( {\Delta F/F_0} \right)$. We considered each neuron to be active for two consecutive frames at the peak of each detected Ca^2+^ transient (to account for the typical Ca^2+^ indicator rise time).

### Electrophysiology in the subiculum and thalamus

We obtained published electrophysiology recordings^30^ (*N* = 2 mice; 62 and 41 cells per mouse) from multiple anterior thalamic nuclei, mainly the anterodorsal nucleus (ADn), and subicular areas, mainly PoS, in freely moving mice foraging for food in an open environment (53 × 46 cm). The authors recorded at 20 kHz, simultaneously from 64 to 96 channels and processed the raw data to extract the LFPs and detect spikes.

### Obtaining the data points

For Ca^2+^ imaging data, we constructed a binary activity matrix of size *N* × *K*, where *N* is the number of neurons and *K* is the number of frames in the movies tracking Ca^2+^ dynamics. If the *i*th neuron was active in the *j*th frame, the *i*th by *j*th element of the matrix was set to 1; otherwise, it was set to 0. The median $\Delta F/F_0$ values per active bin were 0.0229 (5–95%:  0.0082–0.1123) for CA1 and 0.0528 (5–95%: 0.0266–0.1078) for ACC. We then defined each data point as a frame-level binary activity vector of length *N*, namely, a row of the activity matrix. To further analyze the data, we used only frame-level activity vectors with > 1 active neurons (non-zero elements).

For electrophysiology data, we first binned the activity of the neurons using a 100 ms time bin. We then constructed a binary activity matrix of size *N* × *K*, where *N* is the number of neurons and *K* is the number of time bins. If the *i*th neuron fired at least one time during the *j*th time bin, the *i*th by *j*th element of the matrix was set to 1; otherwise, it was set to 0. The median number of spikes per active bin was 2 (5–95%: 1–8). We then defined each data point as a bin-level binary activity vector of length *N*, namely, a row of the activity matrix. For dimensionality reduction and further analysis of the data, we discarded activity vectors with <15 active neurons.

### Dimensionality reduction

Non-linear dimensionality reduction techniques enable the identification of sets of activity patterns that lie in a low-dimension manifold within the high dimension of the data (*N*, number of neurons), even if this manifold is non-linear (Supplementary Figs. 1 and 2). To this end, we used Laplacian Eigenmaps (LEM) for non-linear dimensionality reduction as previously described^21^. In general, such techniques utilize the local relationships between proximal data points to reconstruct a global distance metric. Given *K* data points *x*_1_,…, *x*_*K*_ (number of frames) lying in an *N*-dimensional space of neuronal activity, we constructed a weighted graph with *K* nodes, one for each point, and a set of edges connecting neighboring data points. We considered nodes *i* and *j* to be connected by an edge if *i* is among the *p* nearest neighbors of *j*, or *j* is among the *p* nearest neighbors of *i* (*p* = 0.25–0.5% for all data sets). We used the parameter free (“simple minded”) method for choosing the weights, i.e.,*W*_*ij*_ = 1 if nodes *i* and *j* are connected, and 0 otherwise^21^. We then computed the eigenvalues and eigenvectors for the generalized eigenvector problem: *Lf* = *λDf*, where *D* is the diagonal weight matrix, its entries are column sums of *W*, and *L* = *D* – *W* is the Laplacian matrix. We left out the leading eigenvector (as previously described^21^) and used the next 10 eigenvectors for embedding in a 10-dimensional Euclidean space. The LEM was performed twice. For the second iteration, performed on the reduced dimensional data, we defined nodes *i* and *j* as connected if *i* is among the *p* nearest neighbors of *j*, or *j* is among the *p* nearest neighbors of *i* (*p* = 7.5–15% for all data sets). For further analysis, we used the three leading eigenvectors (after leaving out the first eigenvector). LEM was applied to all data sets. To demonstrate that the distributions of data points in the reduced dimensional space are not obtained by chance, we performed the same analysis for ‘time shuffled’ (Supplementary Fig. 3, middle column) and ‘cell shuffled’ (Supplementary Fig. 3, right column) versions of the data. Time shuffle was obtained by shuffling the times of neuronal activity independently for each cell while maintaining the overall number of active time bins per cell. Cell shuffle was obtained by shuffling the identity of the active cells for each time bin while maintaining the overall number of active cells per time bin.

### Estimation of data internal dimensionality and topology

To estimate the internal dimension of the data, for any given data point in the reduced space, we calculated the number of neighbors within a sphere surrounding it as a function of the sphere’s radius. We used the slope of the number of neighboring data points within a given radius on a log–log scale to estimate the dimension of the data^24^. A simulation illustrating this procedure is presented in Supplementary Fig. 7.

To estimate the topology of the data, we calculated the numbers of components (*β*_0_), holes (*β*_1_), and spaces (*β*_2_), as a function of the radius threshold, using a previously established algorithm^33^ (Javaplex: A research software package for persistent (co) homology, https://github.com/appliedtopology/javaplex). We then searched for components, holes, and spaces that were stable across a wide range of radii. A simulation illustrating this procedure is presented in Supplementary Fig. 18. Since the algorithm is computationally demanding and is not scalable to large data sets, we applied the algorithm to a representative set of data points. To obtain this representative set, we applied *K*-means clustering on the reduced dimensional data and extracted the centroids of the clusters. To overcome sensitivity to noise, we discarded sparse clusters (<50 data points). To ensure a final number of cluster centroids > 50, we used *K* = 70 for the *K*-means procedure.

### *K*-means clustering

In cases in which the data points are continuously distributed, there is no natural separation of the data points into discrete clusters. Therefore, for parameterization of the encoded variable, we used *K*-means, which yielded segmentation of the data into a discrete set of compact subsets. For electrophysiology data, we used *K*-means (*K* = 8) to segment the data into different clusters.

### Topological clustering and sub-clustering

In cases in which the distribution of data points consists of a discrete set of dense clusters, these clusters can be considered as components in terms of data-topology, and the same algorithm that estimates the number of components (*β*_0_, see the Estimation of data internal dimensionality and topology section above) naturally yields a topological-based clustering of the data points. To utilize the topological approach for data clustering, we focused on large components (>250 data points for clustering and > 50 data points for sub-clustering), and chose a radius that captures the maximal number of stable components (see the Estimation of data internal topology section). We associated each of the data points not assigned to any of the defined components with the cluster that contains its nearest assigned neighbor. This procedure was performed by gradually increasing the radius threshold while maintaining the assignment of previously assigned data points (Supplementary Fig. 4).

### Temporal segmentation of the data

For the Ca^2+^ imaging data from the CA1 and ACC, we used the temporal sequence of clusters to temporally segment the data. Applying a segmentation procedure to the data points allowed us to define the beginning and end of each time segment and, at the same time, reduce the number of short deviations from a given cluster that could result from noise and sparse neuronal activity. To segment the data points, we defined two thresholds: (1) maximal interval between consecutive data points that belong to the same cluster; (2) minimal number of data points within a segment. To optimize the segmentation procedure, we systematically tested different values of the maximal interval threshold and examined the obtained distribution of segment lengths. A suitable threshold should result in a robust distribution (not sensitive to small changes of threshold value) of segment lengths. The distribution of number of data points in a candidate segment was clearly bimodal, which allowed valid segments to be distinguished from noise. We performed segmentation independently for each cluster, enabling data points to belong to a single cluster, multiple clusters, or none of the clusters. In practice, the majority of data points belonged to a segment of only one cluster. After segmenting the data, we constructed an average activity vector by measuring the average activity of each neuron during a given time segment. For each set of segments from the same cluster, we applied PCA to the obtained average activity vectors to expose intra-cluster heterogeneity, corresponding to different segment subtypes within a given cluster.

### Calculating the transition matrix

To capture the temporal relationship between the different network states, we calculated the transition matrix, i.e., the probability of a data point to belong to cluster *i* at time *t* + 1 given that the preceding point belongs to cluster *j* at time *t*. A similar analysis was performed at the segment level, where the transition matrix was defined by the probability of a segment to belong to cluster *i* given that the preceding segment belongs to cluster *j*.

### Temporal ordering of clusters and sub-clusters

For the Ca^2+^ imaging data from the CA1 and ACC, we examined the internal structure of the data within a given cluster or segment subtype. We sub-clustered the reduced dimensional data points using topological clustering as described above. To evaluate the temporal ordering of the sub-clusters, for each possible ordering (i.e., permutation), we calculated the sum of probabilities of moving from each sub-cluster to its consecutive sub-cluster. The ordering $\hat I$ of the *M* sub-clusters was set as the order that maximizes the sum of probabilities out of the *M*! possible permutations.

2 $$\hat I ={\mathrm{argmax}}_I\left( {\mathop {\sum}\nolimits_{i = 1}^{M - 1} {P_{I_i,I_{i + 1}}} } \right)$$

Since examining all the possible permutations is computationally demanding, we first calculated the number of times each sub-cluster appeared at the beginning and at the end of a segment. The analysis revealed that one sub-cluster was prominent at the start of a segment and another sub-cluster was prominent at the end of the segment, implying a specific directionality for the ordering of the sub-clusters. This allowed us to constrain the first and last sub-clusters among the *M* sub-clusters. Consequently, we had to examine only (*M* − 2)! possible permutations rather than *M*!.

For electrophysiology data from the ADn and PoS, due to the observed ring topology of the reduced dimensional data (Supplementary Fig. 18) and the trajectory within it (Supplementary Fig. 17a,b), we sought to cyclically order the obtained *M* clusters. To this end, for each ordering, we calculated the sum of probabilities for moving from a cluster to its neighboring clusters from both directions. The cyclical ordering of sub-clusters was set to maximize the sum of these probabilities, out of the (*M* − 1)!/2 possible orders (due to rotation and reflection symmetry). The ordered states of neuronal activity allowed us to reconstruct the encoded variables and calculate the internal tuning curve for each cell.

### Reconstruction of the encoded variables

To reconstruct the variables encoded within the network activity, we parameterized the network states. For Ca^2+^ imaging data from the CA1, we focused on the reconstruction of an internal position within clusters associated with locomotion. We used the obtained sub-clusters and their ordering and assigned evenly spaced internal positions to the different sub-clusters. For comparison with the external position, we set the reflection degree of freedom (out of the two possible orientations of the linear track due to its symmetry) by minimizing the mean squared error between the internal reconstruction and the external variable (performed globally for the entire data set). The error (mismatch) between the estimated position (or phase), based on the reconstructed internal representation $\hat x$, and the actual position (or phase), *x*, was defined as $\hat x - x$.

For electrophysiology data from the ADn and PoS, we reconstructed the internal angle based on the clustering of the entire data set and the obtained cyclical ordering of the clusters. Data points that belong to cluster *k* were assigned an internal angle of $\frac{{2{\mathrm{\pi}}k}}{M}$, where *M* is the number of clusters. We then used a truncated Gaussian kernel W (*σ* = 2 frames, size = 5 frames) to temporally smooth the data. For comparison with the head direction, we set the rotation and reflection degrees of freedom (due to the symmetries of a ring) by minimizing the mean squared error between the internal reconstruction and the external variable (performed globally for the entire data set). The mismatch between the estimated head direction based on the reconstructed internal representation, $\hat \theta$, and the actual head direction, *θ* (or the decoded head direction during sleep; see the REM-sleep decoder section), was defined as ${\mathrm{mod}}\left( {\hat \theta - \theta + \pi ,2\pi } \right) - \pi$.

### Calculation of internal tuning curves

We sought to calculate the tuning curve for each cell relative to the state of the network, rather than relative to any external variable (e.g., position). For this calculation, we first measured the time the network spent (occupancy) at any given state and the number of neuronal activity events within each network state (Ca^2+^ event number or number of spikes). We then divided the number of neuronal activity events by the occupancy, obtaining the activity rate as a function of the internal state of the network—hence the internal tuning curve.

For Ca^2+^ imaging data from the CA1 and ACC, we focused on the reconstruction of internal tuning curves within clusters associated with locomotion. Internal tuning curves were calculated for cells that displayed > 5 Ca^2+^ events within the relevant clusters. For comparison with external tuning curves, after we calculated the occupancy and Ca^2+^ event number vectors, we interpolated the Ca^2+^ event number and the occupancy at the ordered network states (sub-clusters) to increase the number of states so it will match the number of spatial bins. We then used a truncated Gaussian kernel (*σ* = 1.5 bins, size = 5 bins, as used for calculating external tuning curves) to smooth the two functions. Finally, we computed the internal tuning curve for each neuron by dividing the smoothed map of Ca^2+^ event numbers by the smoothed map of occupancy.

For electrophysiology data from ADn and PoS, the internal tuning curves were calculated based on the clustering of the entire data set and the obtained cyclical ordering of the clusters. Data points were smoothed as described above (see the Reconstruction of the encoded variables section). We then binned the internal angle into 40 bins of 9° and calculated the occupancy at any given angular bin and the neuron’s number of spikes within each bin. Finally, we computed the internal tuning curve for each neuron by dividing the number of spikes by the occupancy.

### Calculation of external tuning curves

For Ca^2+^ imaging data from the CA1 and ACC, we calculated the tuning of cells to location. We considered periods wherein the mouse ran > 1 cm/s. We divided the track into 24 bins (4 cm each) and excluded the last 2 bins at both ends of the track, where the mouse was generally stationary. We computed the occupancy and the number of Ca^2+^ events in each bin and then smoothed these two maps (occupancy and Ca^2+^ event number) using a truncated Gaussian kernel (*σ* = 1.5 bins, size = 5 bins)^18,38^. We then computed the activity map (event rate per bin) for each neuron by dividing the smoothed map of Ca^2+^ event numbers by the smoothed map of occupancy. For hippocampal data, we separately considered place fields for each of the two running directions on the linear track.

For data recorded from the ACC, we pooled the data from both running directions on the linear track while taking into account the running phase rather than the absolute location. The pooling was performed by flipping the positional indexing of the linear track while the mouse was running in a given direction (this way, a given position on the track while the mouse was running to the right corresponded to the mirror location while the mouse was running to the left).

For the electrophysiology data from the ADn and PoS, we computed the tuning to head direction. We first binned head directions into 40 bins of 9°. We then calculated for each neuron the average firing rate given an angular bin, by dividing the number of spikes in each bin by the occupancy within that bin.

### Place-tuning analysis

For this analysis, we focused only on active cells (≥5 detected events during the running periods in a given direction). For those cells, we computed the spatial information (in bits per event) using the unsmoothed events-rate map of each cell as previously defined^60^:

3 $${\mathrm{spatial}}\;{\mathrm{information}}\; = \;\mathop {\sum}\nolimits_i {p_i \cdot \left( {\frac{{r_i}}{{\bar r}}} \right){\mathrm{log}}_2\left( {\frac{{r_i}}{{\bar r}}} \right)} ,$$

where *p*_*i*_ is the probability of the mouse to be in the *i*th bin (time spent in *i*th bin/total running time), *r*_*i*_ is the Ca^2+^ event rate in the *i*th bin, and *r̄* is the overall mean Ca^2+^ event rate. We then performed 1000 distinct shuffles of animal locations during Ca^2+^ events, accounting for the spatial coverage statistics for the relevant session and running direction, and calculated the spatial information for each shuffle. This yielded the *p*-value of the calculated spatial information relative to the shuffles. Cells with spatial information higher than that of 95% of their shuffles were considered significant place cells. The fraction of place cells in each session was defined as the number of significant cells out of the number of active cells in that session. For the calculation of trajectory-phase tuning, we applied the same calculation while considering the trajectory-phase pooled over both running directions, instead of the position in a given running direction.

The width of the place field was defined as the number of bins in the vicinity of the preferred position with a rate > 0.5 of the maximum event rate across all bins. For trajectory-phase field width, we pooled the data from both running directions on the linear track while taking into account the running phase rather than the absolute location.

In addition, for each cell (≥5 detected events during the running periods in each running direction), we computed its tendency to encode the same position across both running directions (bidirectionality). For this, we used the measure of bidirectionality as previously defined^61^:

4 $${\mathrm{bidirectionality}} = \frac{{2\mathop {\sum }\nolimits_i {\mathrm{min}}(\tilde P_{{\mathrm{right}}}\left( i \right),\tilde P_{{\mathrm{left}}}(i))}}{{\mathop {\sum }\nolimits_i (\tilde P_{{\mathrm{right}}}\left( i \right) + \tilde P_{{\mathrm{left}}}(i))}}$$

5 $$\tilde P_{{\mathrm{right,left}}}\left( i \right) = \frac{{N_{{\mathrm{bins}}}r_{{\mathrm{right,left}}}(i)}}{{\mathop {\sum }\nolimits_i r_{{\mathrm{right,left}}}(i)}},$$

where $\tilde P$_right_(*i*) and $\tilde P$_left_(*i*) are the normalized values of the rate maps in the *i*th bin for right and left running, respectively. To account for trajectory-phase bidirectionality, we also calculated the bidirectionality measure while flipping the order of the bins in one of the running directions.

### Comparison between internal and external tuning curves

For Ca^2+^ imaging data from the CA1 and ACC, we measured the mismatch between the internal and external tuning curves. For the CA1 data, the mismatch was defined as the difference between the internal and external preferred position; for the ACC data, it was defined as the difference between the internal and external trajectory phase. To test the significance of the similarity in the coding properties, we compared the average absolute mismatch with that obtained for 1000 shuffled data sets. For each of the 1000 shuffled sets, we shuffled between the identities of the cells.

For the electrophysiology data from the ADn and PoS, we quantified the angular tuning of each neuron and compared between the internal and external tuning curves. To quantify the angular tuning, we calculated for each angular tuning curve (either to internal angle or to head direction) the Rayleigh vector: $\mathop {\sum }\limits_{k = 1}^N r\left( {\theta _k} \right)e^{ - i\theta _k}/\mathop {\sum }\limits_{k = 1}^N r\left( {\theta _k} \right)$, where *N* is the number of evenly spaced angular bins, $\theta _k = \frac{{2\pi k}}{N}$, and *r*(*θ*) is the firing rate of the neuron given the angle *θ*. The preferred direction of a neuron was estimated by the angle of the Rayleigh vector of its tuning curve, and the directionality (the degree of its tuning to a single direction) was estimated by the absolute value (length) of the Rayleigh vector. For both preferred direction and directionality (Rayleigh vector length), we compared the values obtained for each neuron from the internal and external tuning curves. To further compare the internal and external tuning curves of each neuron, we calculated the Pearson correlation between the two tuning curves. For the visualization of the data in Supplementary Fig. 17e–g, we labeled cells as head-direction cells if they had a Rayleigh vector length > 0.5 and a peak firing rate > 5Hz.

In both cases, in order to compare the internal tuning curves to the external tuning curves, we used the same degrees of freedom as those obtained in the Reconstruction of the encoded variables section.

### Comparison between internal and external information content

To quantify the amount of information between the internal state of the network or the encoded external variable and the activity of a given neuron, we calculated the internal and external information for each cell. For CA1 cells, the external information was the spatial information as described above. For ADn and PoS cells, the external information was defined based on the head direction of the animal (using the same expression that was used to define spatial information for position). To quantify the internal information, we used the relationship between the internal state of the network and the activity of each given cell. For each cell, we applied LEM to the data while excluding this cell from the activity vectors (leave one out) and clustered the data points (using *K*-means) in the reduced space. Internal information was calculated based on the event rate of the neuron during each one of the network states (clusters), using the same expression employed to define spatial information for position. For CA1 cells, LEM, clustering, and internal information were calculated separately for states C_1_ and C_2_ (which corresponded to leftward and rightward running). Only cells with ≥ 5 detected events during the relevant state were included in the analysis. For ADn and PoS cells, LEM, clustering, and internal information were calculated for the binarized version of the data, as described above. To allow an unbiased comparison between the internal and external information, we used eight clusters for the internal state and eight bins for the external state (position or head direction). In addition, since the distribution of time spent in each state differed between the internal and external states, for each cell, we subtracted the average information obtained from 100 shuffles of that cell.

To quantify the quality of the identification of network states as a function of the number of recorded neurons, we systemically changed the number of the neurons used for the analysis by including only random subsets of the recorded population. For each subset, we calculated the internal information between network states and the activity of each of the cells in the rest of the population.

### REM-sleep decoder

For the electrophysiology data from the ADn and PoS, we calculated the ‘virtual trajectory’ of the internal angle during REM-sleep periods as described above (see the Reconstruction of the encoded variables section). Since the actual head direction is constant during sleep, the internal angle was compared with the ‘virtual trajectory’ of head direction obtained by a maximum likelihood decoder. The maximum likelihood decoder for virtual head direction during REM sleep was based on the external tuning curves during wake periods, using only head-direction cells (>0.5 Rayleigh vector length and > 5 Hz peak firing rate). For comparison between the internal angle and the decoded head direction, we sought the rotation and reflection degrees of freedom (due to the symmetries of a ring) that minimize the decoding mean squared error.

### Sleep-to-wake decoder

We trained a decoder during periods of REM sleep and tested it during periods of awake behavior. The internal angle and internal tuning curves were first calculated for REM-sleep data as described above (see the Calculation of internal tuning curves section) and then used to train a maximum likelihood decoder (Fig. 4f). Next, the decoder was tested on neuronal activity from awake periods. For comparison of the decoded head direction with the actual head direction, we sought the rotation and reflection degrees of freedom (due to the symmetries of a ring) that minimize the decoding mean squared error (inset of Fig. 4f). The decoding error was compared with that obtained for shuffled data (see the Shuffle test section).

### Across-mice decoder

Across-mice decoders rely on the analogy between the internal structures of neuronal activity across different mice (or different days of the experiment) for the same brain region. These decoders were used to infer the external state (position or head direction) of one mouse based on the relations between the internal state (neuronal activity patterns) and external state of another mouse (or of the same mouse at another day of the experiment). We used the neuronal activity within the reduced dimensional space to parameterize data points within the internal state. Specifically, for the Ca^2+^ imaging data, clusters and ordered sub-clusters therein were translated into positions on a 1D segment. For electrophysiology data, we used the circular order of the clusters to assign an angle to each data point. Based on this parameterization, the mapping across network states within internal structures obtained for different mice (or different days for the same mouse) was defined. In all cases, the mapping was defined up to the internal symmetry of the internal structure: rotation and reflection for the PoS and ADn data and reflection for the CA1 data. For mouse 1, neuronal activity at each time frame was associated with a specific network state. Based on the similarity between internal structures, an analogous network state was found in mouse 2. This network state of mouse 2 was associated with a known external state. The decoded external state of mouse 1 was set as the same associated external state of mouse 2. The external state decoding error was calculated and compared with that obtained for shuffled data (see the Shuffle test section).

### Trajectory-phase decoder

For Ca^2+^ imaging data recorded from the ACC, we tested the similarity between neuronal activity during running in one direction of the linear track and the neuronal activity during running in the other direction. We first defined the trajectory phase as the distance of the mouse from the starting point, namely, the distance from the left edge when running rightward and the distance from the right edge when running leftward. We then constructed a decoder that estimates the trajectory phase in a given direction (test data) based on the relations between neuronal activity pattern and trajectory phase in the other direction (training data). Specifically, for each data point obtained during running in a given direction, we found its nearest neighbor in the reduced space of neuronal activity among all data points obtained during running in the opposite direction. The decoded phase was set as the animal’s trajectory phase at the time of the nearest neighbor data point. The trajectory phase decoding error was calculated and compared with that obtained for shuffled data (see the Shuffle test section).

### Trajectory phase, time, and speed by acceleration encoding

Since the behavior in the linear track is stereotypic, there is a high correlation between trajectory phase, time from the beginning of a track traversal, and the speed and acceleration of the animal. Therefore, we turned to examine which of these external variables best explain the activity of ACC neurons. For each cell with ≥10 detected events during running, we calculated event rates for each combination of trajectory phase and time from the beginning of a track traversal (using 20 bins for each variable). We then calculated for each trajectory phase the standard deviation of event rates across different times, and then calculated the weighted average of the standard deviation across all phases (SD_T_—a measure of the variability in the time in which the cell is active). Similarly, we calculated for each time the standard deviation of event rates across different phases, and then calculated the weighted average of the standard deviation across all times (SD_P_—a measure of the variability in the trajectory phase in which the cell is active).

In addition, we devised decoders of trajectory phase, time from the beginning of a track traversal, and speed by acceleration, based on population activity. The decoders were trained on the activity patterns from half of the data and tested on the other half (odd versus even minutes from the recording). For the binning of the trajectory phase, we excluded 8 cm from each side of the track (where the mice tend to be stationary) and divided the track into 20 evenly spaced spatial bins. We then considered both running directions, while flipping the order of spatial bins between them, which yielded 20 trajectory phase bins. For the binning of time from the beginning of a track traversal, we divided the time from the beginning of track traversal to the maximal temporal length of a trajectory into 20 evenly spaced time bins. Within a track traversal, mice typically accelerate up to the maximal speed (speeding up segment—positive egocentric acceleration) and then keep on running while slowing down until they reach the end of the track (slowing down segment—negative egocentric acceleration). Therefore, for the binning of speed by acceleration, we divided the speed during the speeding up segment from 0 to 40 cm/s into 10 evenly spaced bins, and the speed during the slowing down segment from 40 to 0 cm/s into 10 additional evenly spaced bins. This yielded a total of 20 speed-by-acceleration bins, ordered according to the typical behavioral trajectory within a track traversal. For each trajectory phase, time, or speed-by-acceleration bin (20 bins for each decoder), the activity vectors were averaged across the entire training set, yielding 20 vectors, each corresponding to a given bin. Then, for each activity vector in the test set, the decoded trajectory phase, time, and speed by acceleration was set as the bin with which it had the highest Pearson correlation.

Finally, we calculated the mean squared error for all decoders over all activity vectors in the test data and the explained variance, which was obtained by

6 $${\mathrm{Explained}}\;{\mathrm{variance}} = 1 - \frac{{{\mathrm{mean}}\;{\mathrm{squared}}\;{\mathrm{error}}\;{\mathrm{(data)}}}}{{{\mathrm{mean}}\;{\mathrm{squared}}\;{\mathrm{error}}\;{\mathrm{(shuffle)}}}} \cdot$$

The mean squared error for shuffled data was obtained by shuffling the labels (bins of trajectory phase, time, or speed by acceleration) of the test data set while maintaining the overall probabilities of each label.

### Shuffle test

To test the significance of decoding accuracy, we measured the difference between the decoded values and the measured behavior at each time bin. We then compared the performance of each of the decoders described above to the performance obtained for 1000 shuffled data sets. For each of the 1000 shuffled sets, we shuffled the decoded values across the different time bins and set the degrees of freedom (rotation and reflection for angle, and reflection for position) to those that minimized the decoding mean squared error (done separately for each shuffle).

### Labeling of behavioral states

For Ca^2+^ imaging data from the CA1 and ACC, we manually labeled the behavior of the mouse. To identify periods corresponding to behavioral states of ‘Running’, ‘Drinking’, ‘Turning’, and ‘Rearing’, we analyzed the movies from the overhead camera that tracked animal behavior using video tracking software (EthoVision XT 11.5). We manually tagged the beginnings and ends of each behavioral state throughout each movie. Further separation of the running state to the start and end of running was done based on animal position along the linear track: ‘Start run’ and ‘End run’ were defined from the starting point to the center of the linear track, and from the center to the end point of the track, respectively. This behavioral labeling was used both for the comparison with neuronal clustering (Fig. 2f) for the calculation of correlations between representations of behavioral states (Supplementary Fig. 15), and for the analysis of code and structure stability over time (Supplementary Fig. 20).

### Code stability and structure stability

For the analysis of code stability and structure stability we used the manual labeling of behavioral states. For the analysis of code stability, we calculated the ensemble activity vectors for each behavioral state (drinking at edge 1, turning at edge 1, running, turning at edge 2, and drinking at edge 2), separately for each imaging day. Ensemble activity vectors were defined as the mean event rate for each neuron in a given behavioral state and imaging day. We then calculated the Spearman correlations between the ensemble activity vectors of the same behavioral state at two different days. For each pair of imaging days, we calculated the weighted average of the correlations across the different behavioral states (weighted according to the portion of time spent in each state). For the analysis of structure stability, we calculated for each behavioral state in each imaging day, the center of mass of data points in the reduced dimensional space of neuronal activity (dimensionality reduction was applied separately to the data from each day). We then calculated for each day the (Euclidean) distance matrix across the centers of mass of the different states. For each pair of days, we calculated the Spearman correlation between the two distance matrices. Since the distance matrix is symmetric and the values on the main diagonal are zero by definition, we considered only the entries above the main diagonal.

For both measures (code correlation and structure correlation), we calculated the average normalized correlation as a function of elapsed time, and normalized the values to the correlation at the minimal elapsed time (2 days). Due to the symmetry of the linear track to reflection, we considered for each pair of days the maximal correlation among the two possible orientations. We then fit a linear function to each curve (average code correlation and average structure correlation versus elapsed time) and calculated the slope and the 95% confidence interval. Since the neural code for turning and rearing at the same position was similar (Supplementary Fig. 15b), we considered both behaviors as the same network state.

The significance of the stability across each pair of days was obtained by shuffling the identity of the behavioral states in one of the 2 days, and calculating the correlation between the distance matrices for each shuffle. Due to the symmetry of the linear track to reflection, we considered for each pair of days the higher correlation among the two null permutations, corresponding to the two possible orientations of the track. We then calculated the *p*-value based on the rank of the correlation for the null permutation among all possible orientations.

### Statistical analysis

Statistical analysis was conducted using MATLAB 2016b software. One-tailed unpaired *t*-test with Holm–Bonferroni correction for multiple comparisons was conducted for between-region comparisons of activity correlation level.

### Reporting summary

Further information on research design is available in the Nature Research Reporting Summary linked to this article.

## Supplementary information

Supplementary Information

Reporting Summary

Description of Additional Supplementary Files

Supplementary Movie 1

Supplementary Movie 2

Supplementary Movie 3

Supplementary Movie 5

Supplementary Movie 4

Supplementary Movie 6

## Data Availability

The data that support the findings of this study are available from the corresponding author upon reasonable request.

## Code availability

The analysis code used in this study is available at https://github.com/zivlab/island.
