# Supplementary material for: Revealing neural correlates of behavior without behavioral measurements
Source: Nat Commun. 2019 Oct 18;10:4745. doi: 10.1038/s41467-019-12724-2 (PMC6802184; doi:10.1038/s41467-019-12724-2)
Supplement: Supplementary file 1 — Supplementary Information [file 41467_2019_12724_MOESM1_ESM.pdf]

Supplementary Information

Revealing neural correlates of behavior without behavioral measurements

Alon Rubin, Liron Sheintuch, Noa Brande-Eilat, Or Pinchasof, Yoav Rechavi, Nitzan Geva, and Yaniv Ziv

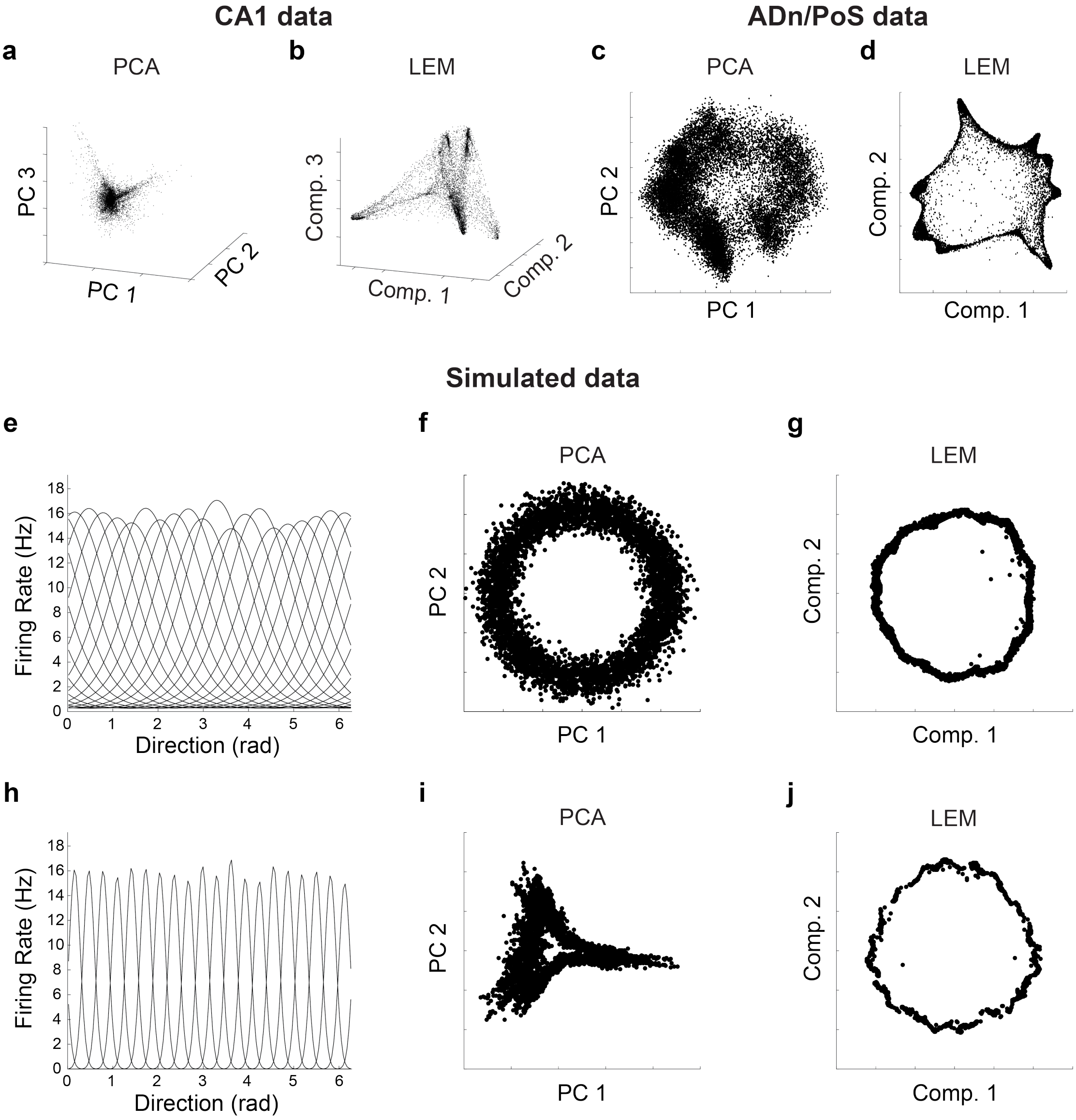

Supplementary Fig. 1 Non-linear dimensionality reduction enables more accurate estimation of the internal structure of neuronal activity than linear methods. **a-b** Ca^{2+} imaging data: The distribution of data points in the reduced dimensional space of neuronal activity, using PCA **a** and LEM **b**. **c-d** Electrophysiological data: The distribution of data points in the reduced dimensional space of neuronal activity, using PCA **c** and LEM **d**. **e**-**h** We simulated neuronal responses for 20 conditionally independent neurons that follow Poisson statistics. The centers of the tuning curves were equally spaced on a one-dimensional circular variable and were either widely tuned **e** or narrowly tuned **h**. **f-g** The distribution of data points in the reduced space of neuronal activity, using PCA **f** and LEM **g**, for widely tuned neurons. Both methods captured the ring topology of the data. **i-j** The distribution of data points in the reduced space of neuronal activity, using PCA **i** and LEM **j**, for narrowly tuned neurons. LEM exposed the ring topology more successfully than PCA.

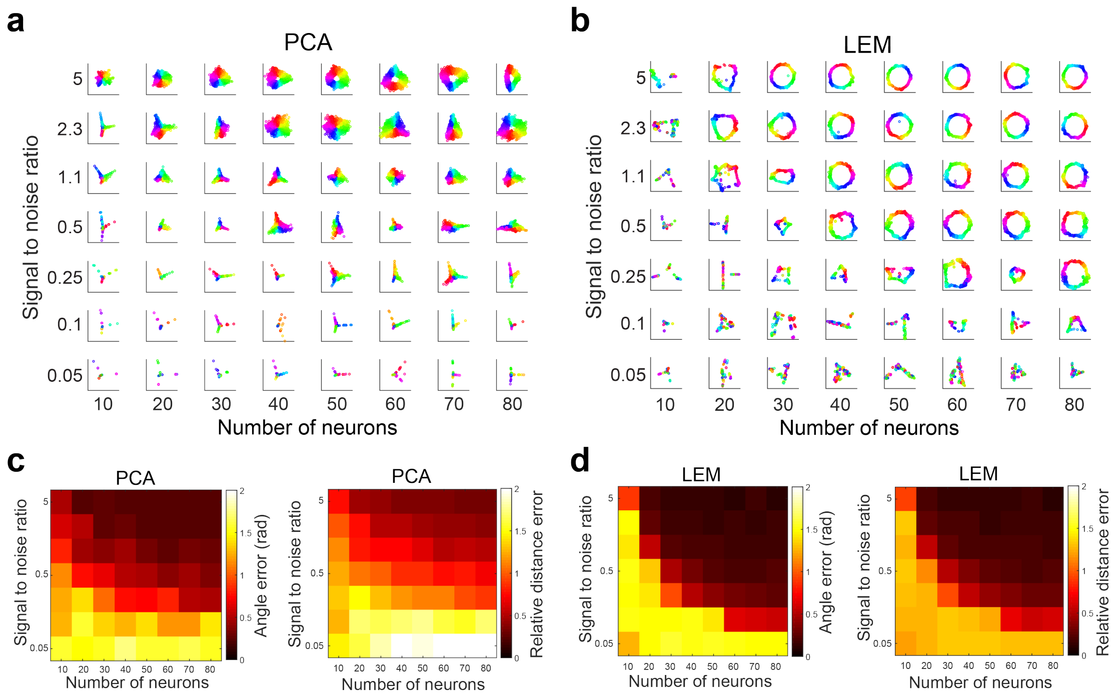

Supplementary Fig. 2 Non-linear dimensionality reduction requires less neurons and lower firing rates for accurate estimation of the internal structure compared to linear methods. **a-d** We simulated neuronal responses for 10-80 conditionally independent neurons that follow Poisson statistics. The centers of the tuning curves were equally spaced on a one-dimensional circular variable. **a-b** The distribution of data points in the reduced space of neuronal activity, using PCA **a** and LEM **b**, for different numbers of neurons and signal-to-noise ratios (SNR). LEM required a smaller number of neurons and SNR to capture the ring topology. SNR was determined by changing the average firing rates of the Poisson statistics. **c-d** The reconstruction error for the angle of each data point within the ring (left) and the relative distance from the fitted position on the ring (right) as a function of the number of neurons and SNR using PCA **c** and LEM **d**. The reconstruction errors are smaller for LEM compared to PCA for most numbers of neurons and SNRs. The tuning widths were set to an intermediate value between the narrow and wide tuning curves in Supplementary Fig. 1e, h.

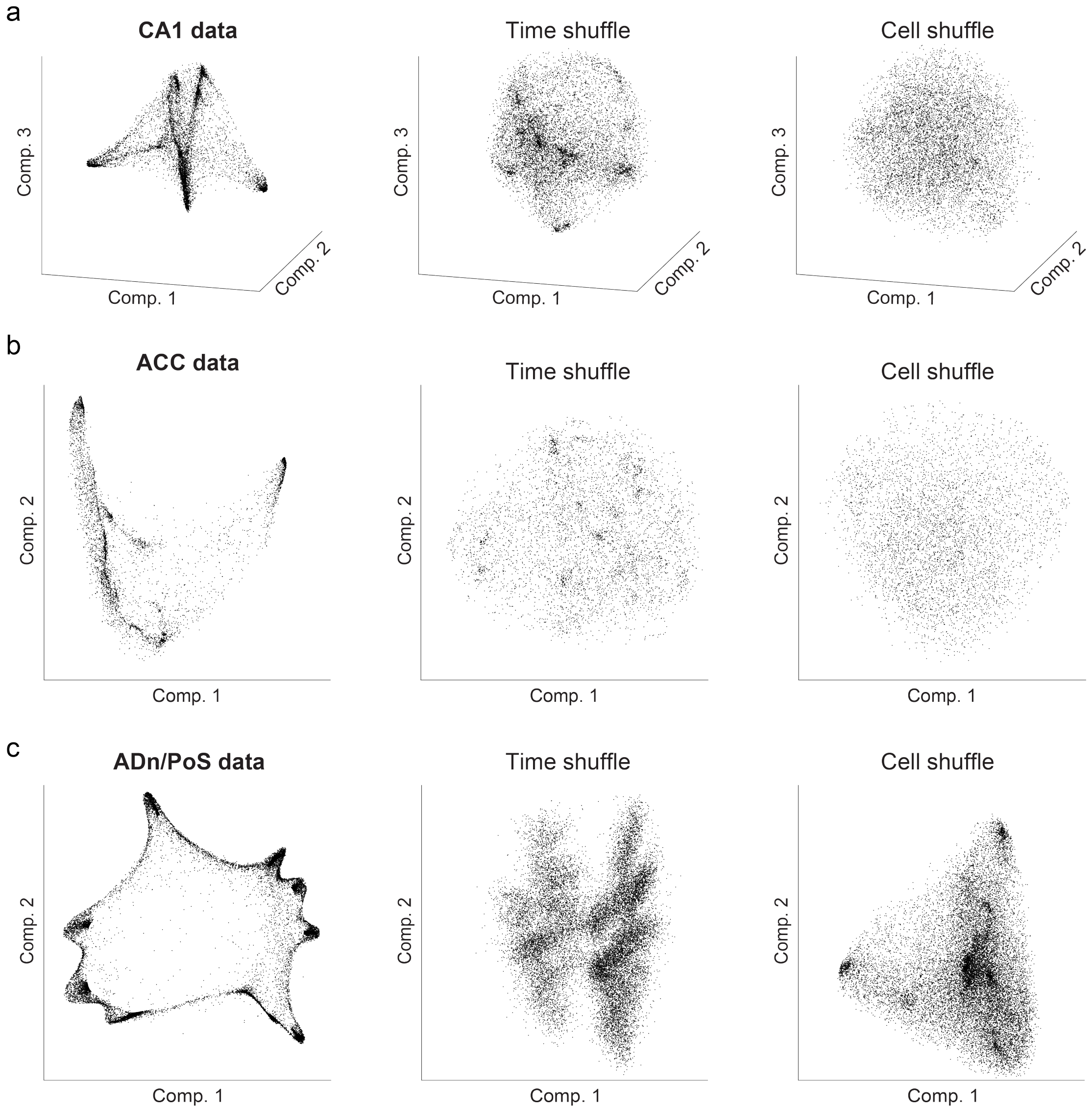

Supplementary Fig. 3 Dimensionality reduction exposes meaningful internal structures in neuronal activity data but not in shuffled data. **a-b** Dimensionality reduction using LEM exposes a small number of dense clusters in neuronal activity data from hippocampal CA1 (**a** - left) and ACC (**b** - left), but shows no clear structure in shuffled data (center and right of **a-b**). **c** Dimensionality reduction using LEM exposes a ring structure in neuronal activity data from the ADn and PoS (left), but shows no clear structure in shuffled data (center and right). The 'time shuffle' was obtained by shuffling the times of neuronal activity separately for each cell; the 'cell shuffle' was obtained by shuffling the identity of the active cells for each time bin.

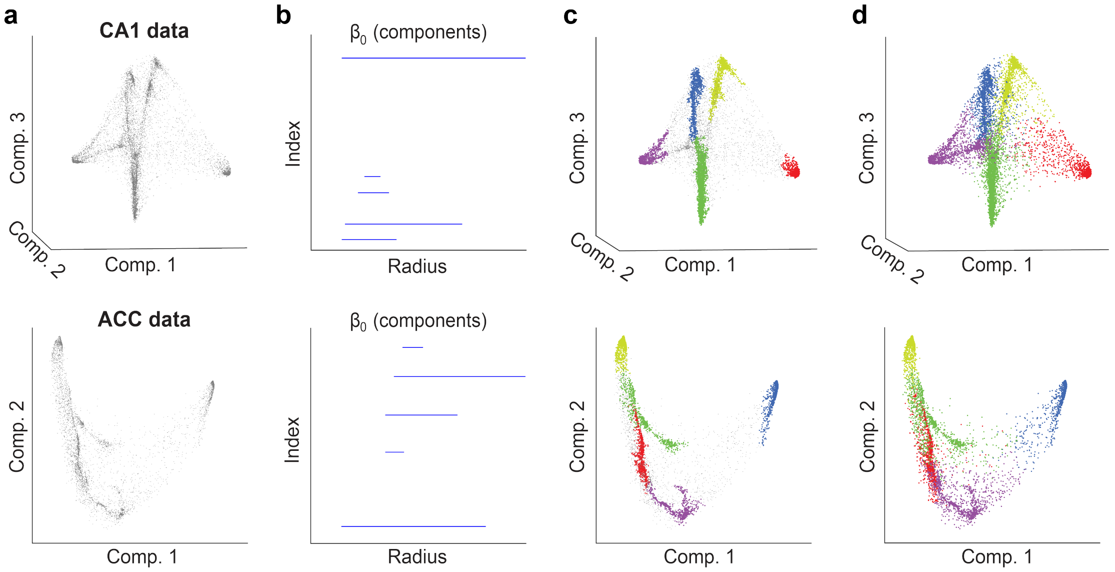

Supplementary Fig. 4 Using topological data analysis to cluster neuronal activity data. **a** The distribution of data points in the reduced dimensional space of neuronal activity for the CA1 (top) and ACC (bottom). **b** The number of separate components (β_0) in the distribution of data points in **a** as a function of the radius threshold. **c** The same data points in **a** colored according to the different components identified in **b**. Data points not assigned to any of the components are shown in gray. **d** By gradually increasing the radius threshold, the remaining data points are assigned to the component of their nearest neighbor (smallest Euclidean distance).

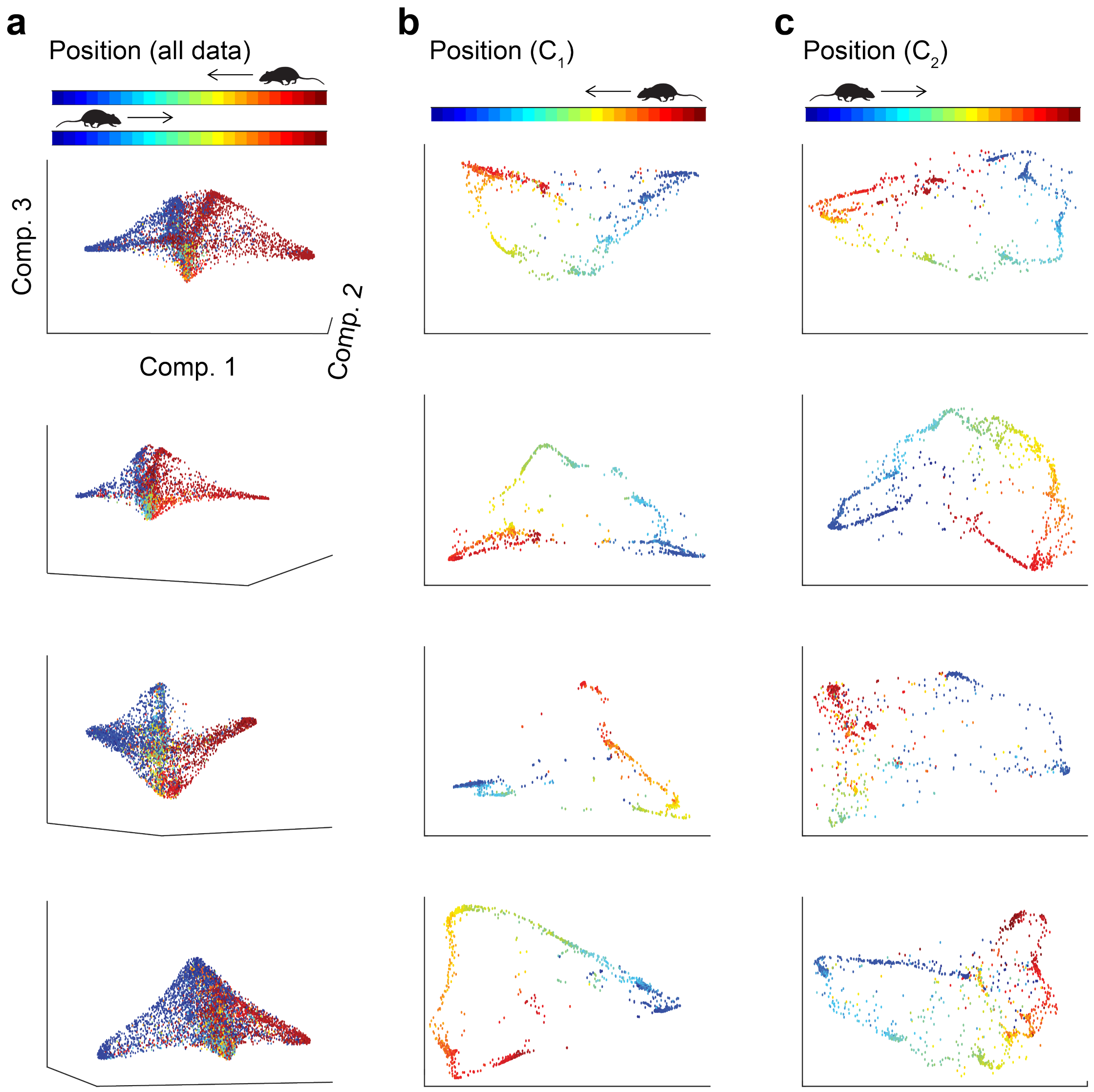

Supplementary Fig. 5 Examples of the internal structures of neuronal activity for CA1 data. **a** The distribution of data points in the reduced dimensional space of neuronal activity for four different mice. Data points are colored according the position of the mouse. **b-c** Dimensionality reduction performed on the activity during epochs in which the network was in state subtype C_1 **b** or C_2 **c** for the same mice shown in **a**. Data points are colored according to the position of the mouse. Data in **b** and **c** were temporally smoothed using a 250 msec rectangular window.

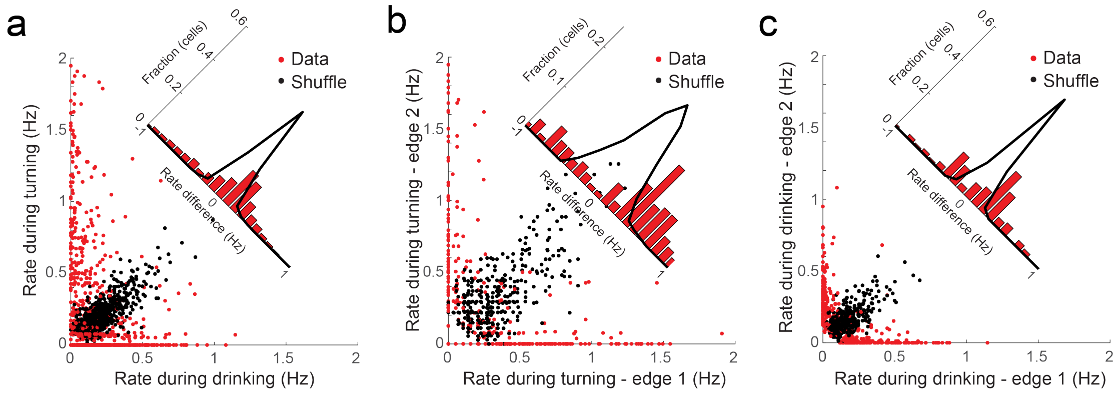

Supplementary Fig. 6 Discrete neuronal states reveal two different behavioral states at the same spatial location. **a** Neuronal activity rates (events/sec) during turning versus during drinking at the same position. Inset, distribution of the rate difference between epochs of turning and drinking. **b-c** Neuronal activity rates (events/sec) during turning **b** or drinking **c** at one end of the track versus the other end of the track. Inset, distribution of the rate difference between the two ends of the track. Data is pooled from N=4 mice.

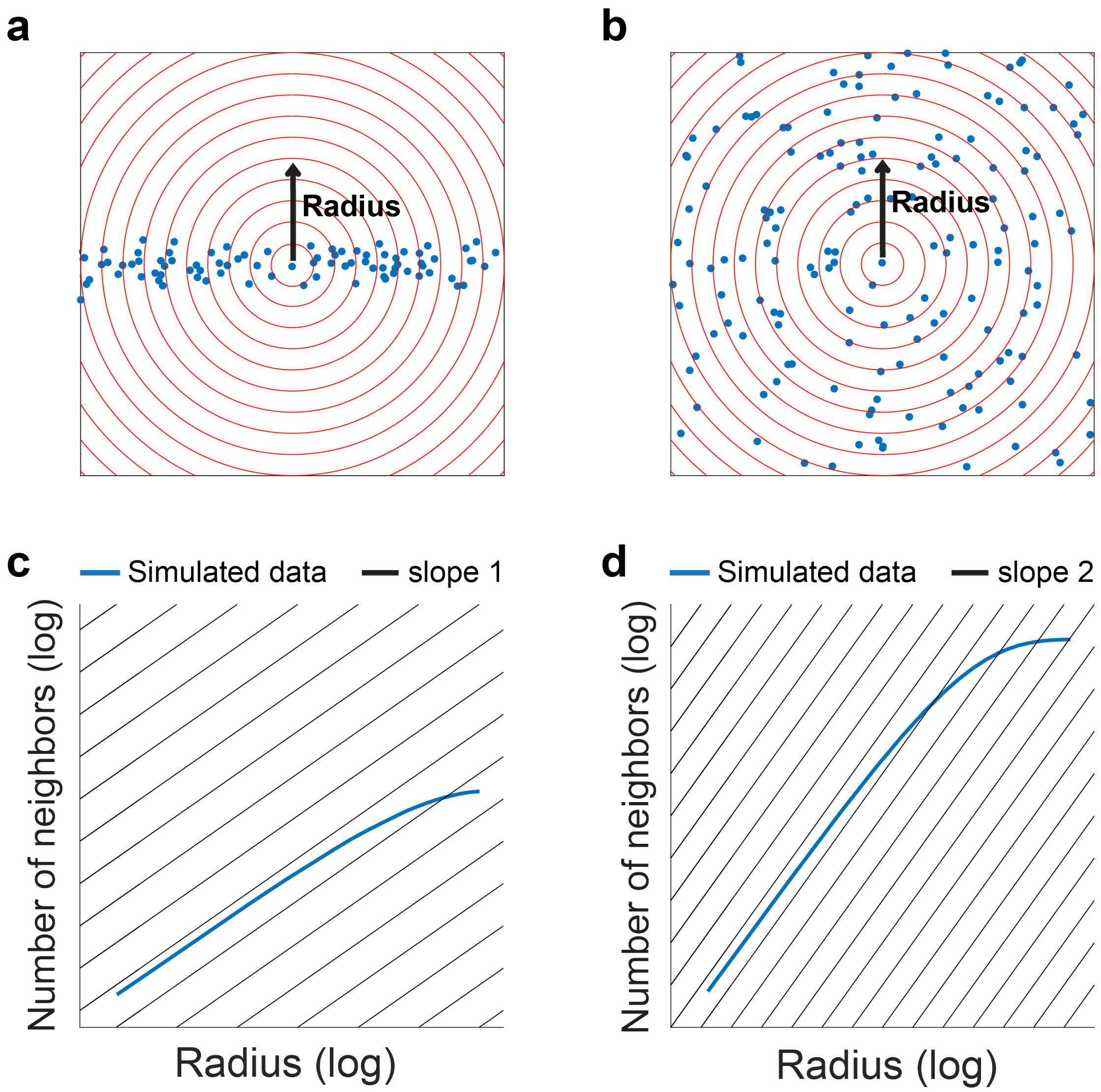

Supplementary Fig. 7 Estimation of the internal dimension of the data. **a-b** Simulated data points, for 1-dimensional data **a** and for 2-dimensional data **b**. The red circles indicate different distances (radii) from a given data point. **c-d** The average number of neighboring data points increases with the radius obeying a power law. The power is indicative of the internal dimension of the data. The number of neighboring data points increases linearly **c** or quadratically **d** with the radius for 1-dimensional and 2-dimensional data, respectively.

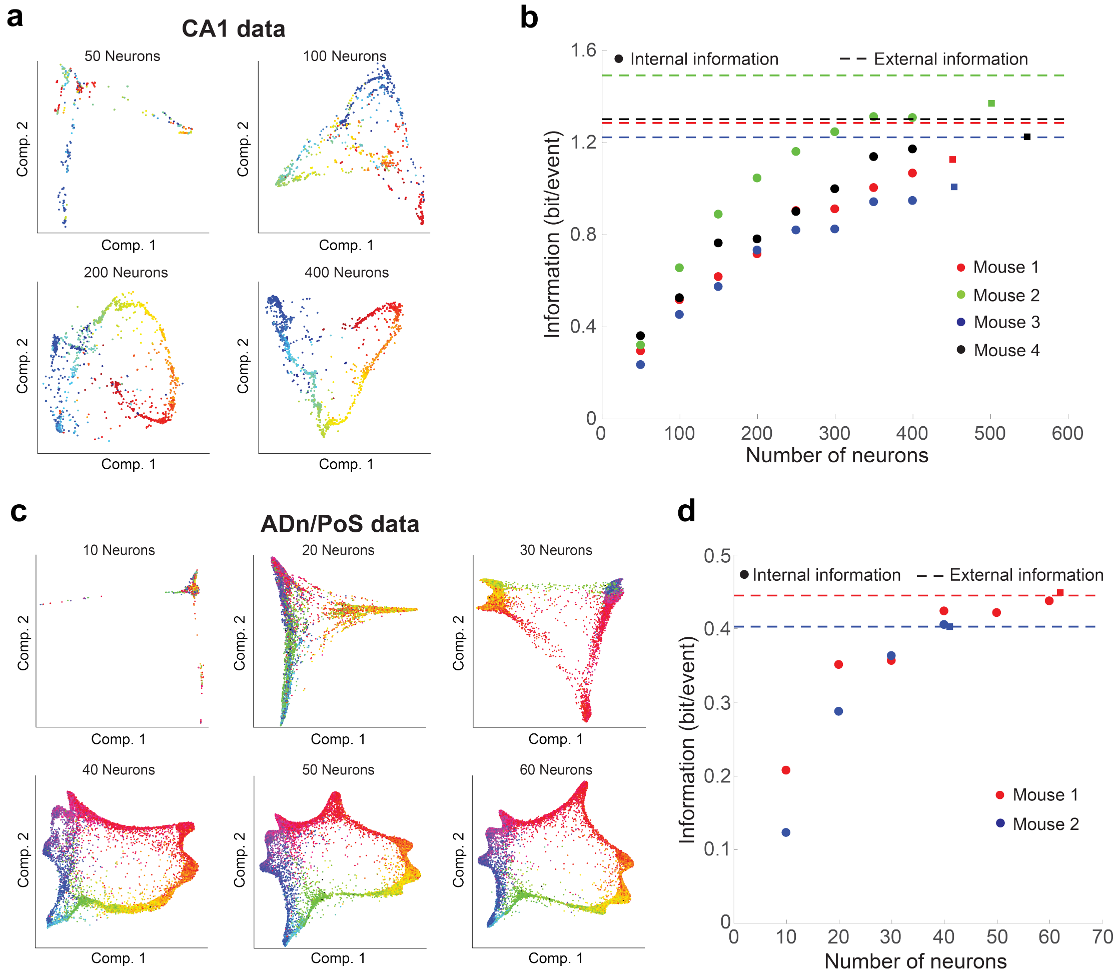

Supplementary Fig. 8 Internal structure of neuronal activity in the CA1 and in the ADn and PoS as a function of the number of neurons. **a** Dimensionality reduction applied to neuronal activity data from the CA1 (state C_2) using different numbers of neurons. Data points are colored according to the mouse position along the linear track. **b** The average information each CA1 neuron carries about the internal network state for different numbers of neurons (colored points; squares show the obtained values when using all the neurons in the data set) and the position along the linear track (dashed lines, independent of the number of neurons). **c** Dimensionality reduction applied to neuronal activity data from the ADn and PoS using different numbers of neurons. Data points are colored according to the head direction. **d** The average information each ADn and PoS neuron carries about the internal network state for different numbers of neurons (colored points; squares show values obtained using all the neurons in the data set) and the head direction (dashed lines, independent of the number of neurons). Calculating the internal information was done by applying the dimensionality reduction analysis to the data, each time leaving out the neuron for which the information was estimated.

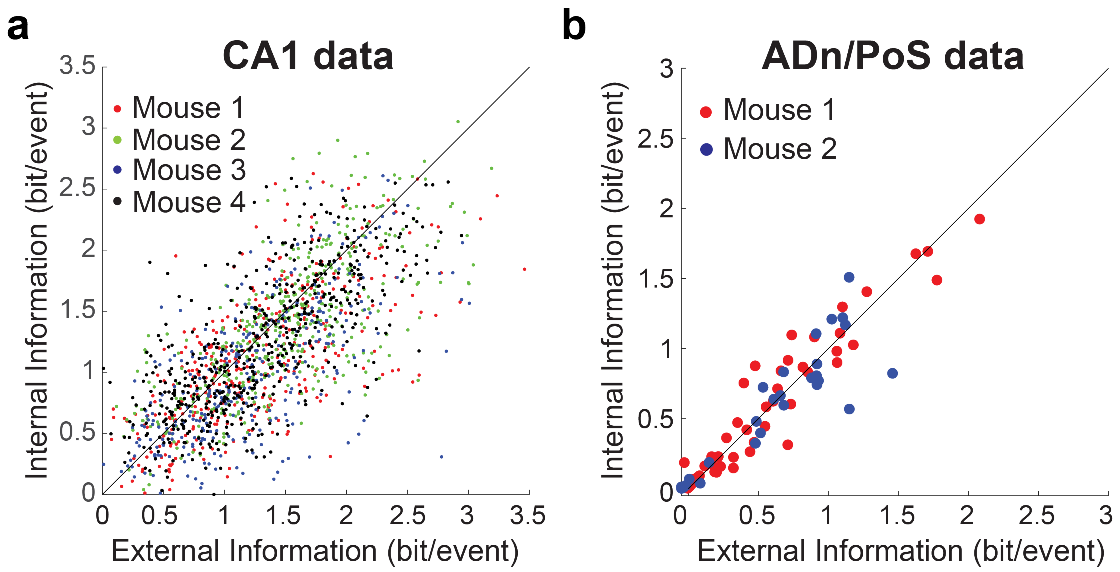

Supplementary Fig. 9 Comparison of the information carried by neurons in the CA1 and in the ADn and PoS about the internal network state or the external variable. **a** The information each CA1 neuron carries about the internal network state (internal information) and the position along the linear track (external information). Internal information was higher than external information in 39% of the cells (144/375, 170/417, 103/331, and 214/517 cells for mice 1-4, respectively) **b** The information each neuron in the ADn and PoS carries about the internal network state (internal information) and head direction (external information). The internal information was higher than the external information in 68% of the cells (42/62 and 28/41cells for mice 1-2, respectively). The internal information was calculated by applying the dimensionality reduction analysis to the data, each time excluding the neuron for which the information was estimated. To correct for sampling bias in the estimation of information, for each cell the average information across all of its shuffles was subtracted.

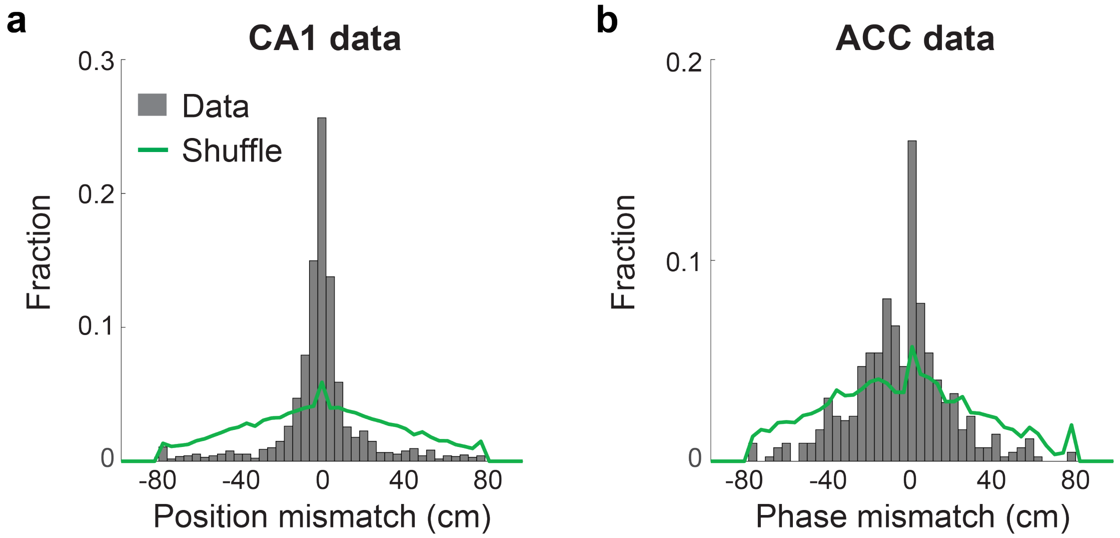

Supplementary Fig. 10 Internal tuning curves of the hippocampal CA1 and ACC reveal similar coding properties to those of their corresponding external tuning curves. **a-b** The distribution of differences in the preferred position **a** and preferred phase **b** between the calculated internal and external tuning curves of the same neurons, for data recorded from the hippocampal CA1 **a** and from the ACC **b**. Data are represented in gray and shuffled data, in green. Data in **a** and **b** was pooled from N=4 and N=3 mice, respectively.

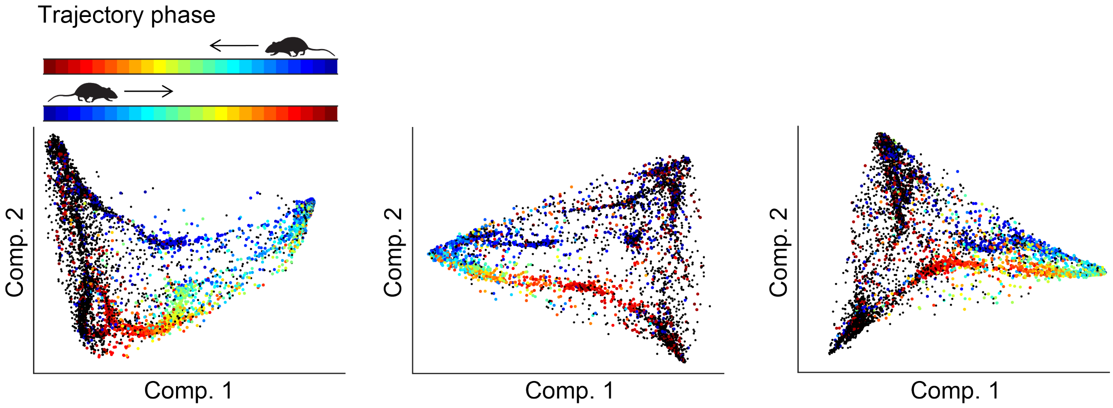

Supplementary Fig. 11 Examples of internal structures of neuronal activity for ACC data. The distribution of data points in the reduced dimensional space of neuronal activity for three different mice. Data points are colored according to the trajectory phase of the mouse.

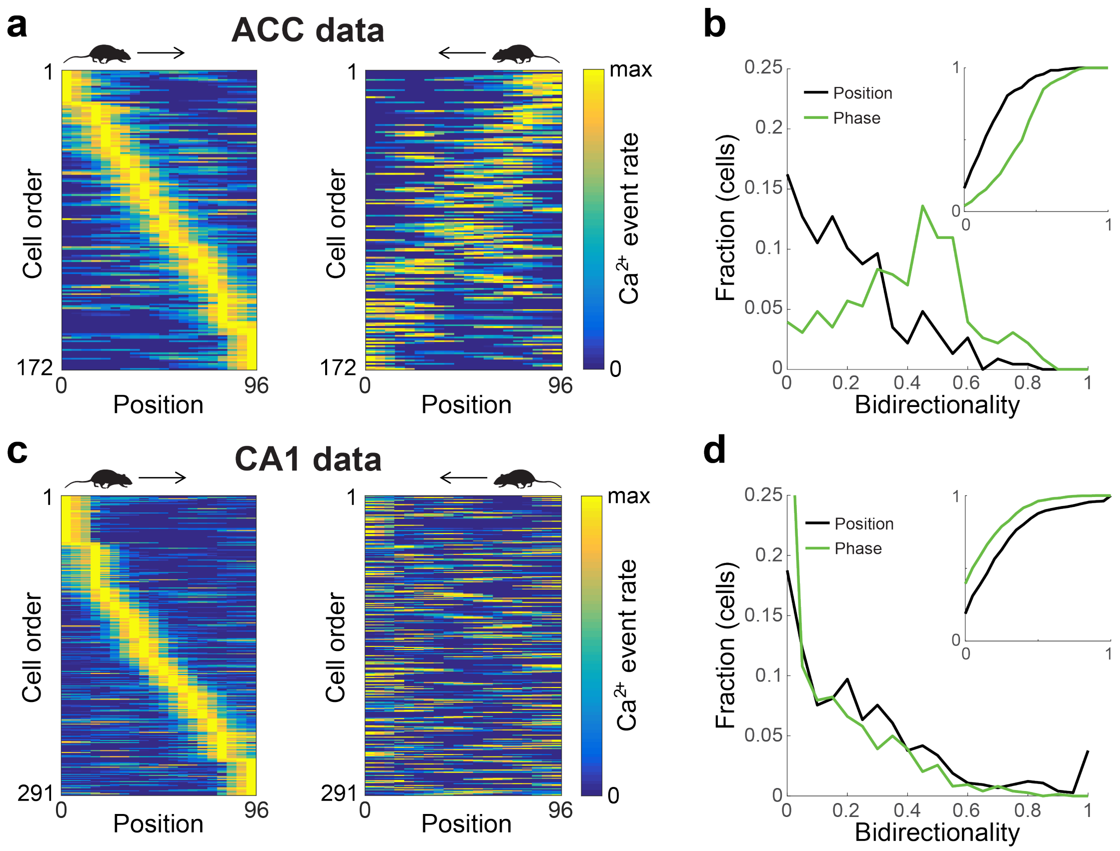

Supplementary Fig. 12 Neurons in the ACC, but not in the CA1, encode the trajectory phase for mice running on a linear track. For each neuron, we calculated its Ca^{2+} event rate as a function of the animal's position (spatial tuning curves) when running in a given direction. **a** Spatial tuning curves for ACC neurons during rightward running (left) and for the same neurons during leftward running (right). For both running directions, the neurons are ordered according to their preferred position along the track during rightward running. **b** The degree to which the cells in the ACC encode the same position (black) or the same trajectory phase (green) across the two opposite running directions. ACC neurons display higher similarity across opposite positions in the opposite running directions (trajectory phase) compared to the same positions (Kolmogorov-Smirnoff, $p=3.5x10^{-23}$). Data in **b** pooled from N=3 mice. **c** Spatial tuning curves for CA1 hippocampal neurons during rightward running (left) and for the same neurons during leftward running (right). For both running directions, the neurons are ordered according to their preferred position along the track during rightward running. Note that while in the hippocampus there is no clear relationship between the preferred positions of the same neurons in the two different running directions, the neurons in the ACC tend to have a symmetrical preferred position for the two directions, hence coding the trajectory phase. **d** The degree to which the cells in the CA1 encode the same position (black) or trajectory phase (green) across the two opposite running directions. CA1 hippocampal neurons display higher similarity across the same positions in the opposite running directions compared to the opposite positions (Kolmogorov-Smirnoff, $p=1.4x10^{-16}$). Data in **f** pooled from N=4 mice.

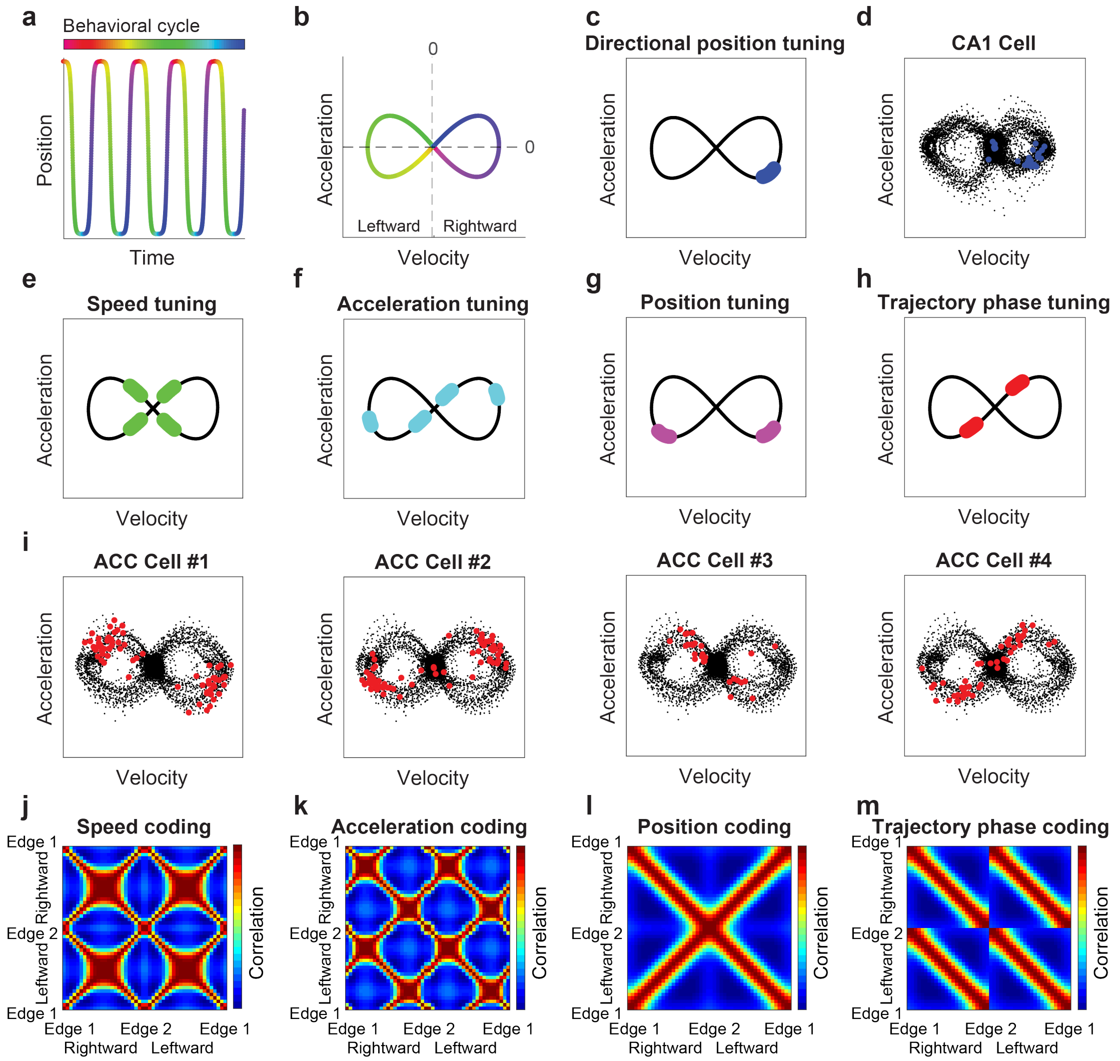

Supplementary Fig. 13 The encoding of trajectory phase in the ACC could not be accounted for by speed or acceleration. **a** A simulated trajectory along a linear track. **b** The acceleration versus the velocity for the trajectory in **a**. **c** The activity of a simulated place cell (blue dots) that is responsive at a particular location in one running direction, overlaid on the velocity-acceleration plot in **b**. **d** The activity of an example place cell from the hippocampal data (blue dots) overlaid on the velocity-acceleration of the mouse (black dots). **e-h** The activity of a simulated cell overlaid on velocity-acceleration plot, for a cell that is responsive at a given speed **e**, acceleration **f**, position **g**, and trajectory phase **h**. **i** The activity of four example cells from the ACC data (red dots), overlaid on the velocity-acceleration of the mouse (black dots). The activity matches the simulated trajectory phase cells. **j-m** Pearson correlation between simulated ensemble activity patterns from different spatial locations on the linear track, for cells that are encoding speed **j**, acceleration **k**, position **l**, and trajectory phase **m**. The ensemble activity (as shown in Fig. 2m) matches the simulated trajectory phase ensemble.

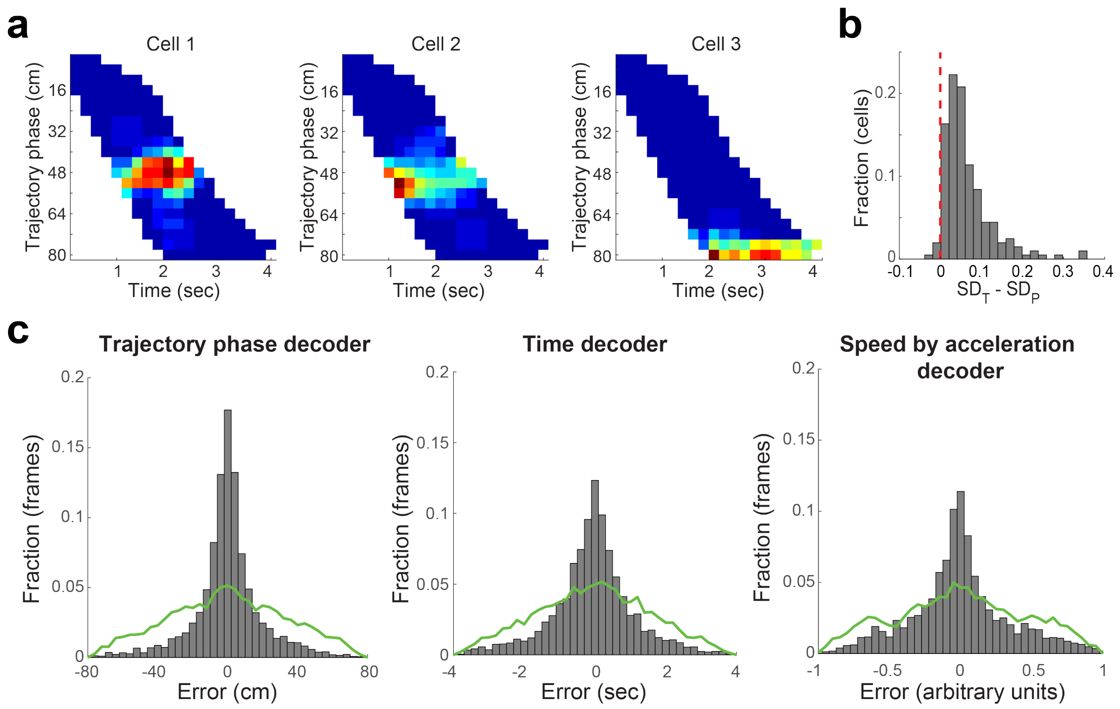

Supplementary Fig. 14 Neuronal activity in the ACC is more accurately explained by trajectory phase than by time and speed. **a** Three example cells that show narrower tuning to trajectory phase compared to time within the trajectory. **b** Cells show higher variability in event rates at their preferred time across different trajectory phases (SD_T) compared to their preferred trajectory phase across different times (SD_P). Data pooled from N=3 mice. **C** Decoding of trajectory phase from neuronal activity (left) is more accurate compared to decoding of time (middle) or speed by acceleration (right). Explained variance = 0.67, 0.48, and 0.40, respectively. Decoder errors (gray) are compared to shuffled data (green).

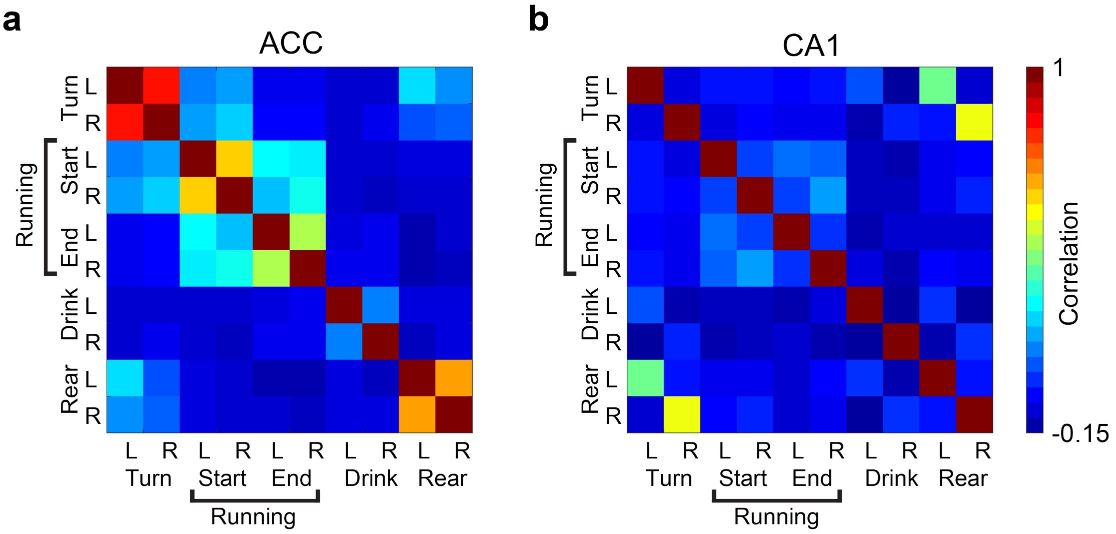

Supplementary Fig. 15 Neuronal activity during epochs of a given behavioral state are similar across opposite sides of the track for data recorded from the ACC, but not for data from the hippocampus. **a-b** Pearson correlation between ensemble activity patterns given the behavioral state and side of the linear track, for the ACC **a** and hippocampal CA1 **b**. Ensemble activity patterns are defined as the mean event rate for each neuron given a behavioral state on the same side of the track. L – left side or leftward epochs, R – right side or rightward epochs. Data averaged over N=3 mice in the ACC and N=4 mice in the hippocampus.

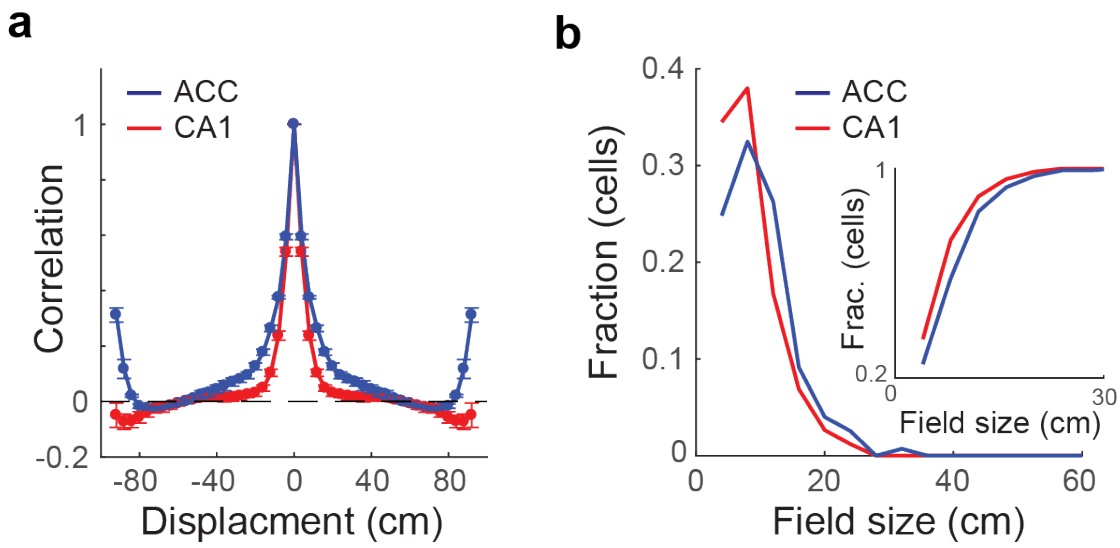

Supplementary Fig. 16 Neuronal representations in the hippocampus are slightly more sharply tuned to position than in the ACC. **a** Pearson correlation (mean ± SEM) between ensemble activity patterns for different spatial displacements of spatial locations on the linear track, for data recorded in the ACC (blue) and the hippocampus (red). Ensemble activity patterns are averaged over mice and over the two running directions. **b** Field sizes are larger in the ACC (blue) than in the CA1 (red). Kolmogorov-Smirnoff, p=0.00012. Average spatial information in the ACC is lower than in the CA1 (1.87±0.09 bit/event for ACC versus 2.24±0.20 bit/event for CA1, two-sided t-test$_{(5)}$, t=-2.87, p=0.035). A smaller percentage of cells are tuned to phase in the ACC compared to position in the CA1 (15.9±0.9% for ACC versus 30.4±5.0% for CA1, two-sided t-test$_{(5)}$, t=-4.87, p=0.039). Data is pooled from N=3 mice in the ACC and from N=4 mice in the CA1.

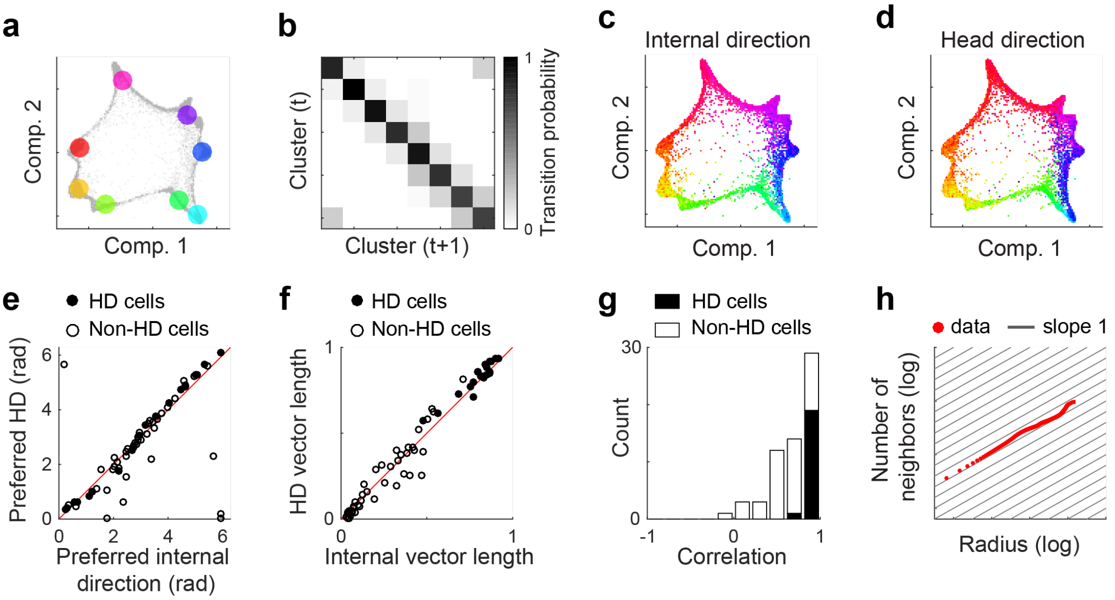

Supplementary Fig. 17 Internal representation of head direction obtained without relying on behavioral data. **a** Clustering of the data in Fig. 4b. The centers of masses of each cluster (colored dots) are overlaid on the data points in Fig. 4b (gray shaded dots). **b** The transition matrix (i.e., the probability of a data point appearing in cluster *i* given that the preceding data point was in cluster *j*) shows that consecutive data points are more likely to be in the same or adjacent clusters. **c-d** The distribution of data points in the reduced dimensional space of neuronal activity, color coded to reflect the reconstructed internal direction **c** and actual head direction **d**. **e-f** The preferred direction **e** and Rayleigh vector length **f** calculated for the internal tuning curves (x-axis) and for the external tuning curves (y-axis). Filled circles indicate cells significantly tuned to head direction. **g** Distribution of correlations between internal and external tuning curves. Cells significantly tuned to head direction are shown in black. **h** Estimation of internal dimension: Cumulative number of neighboring data points as a function of the radius in the reduced dimensionality space, plotted on a log-log scale. The slope of the data (red) is approximately one (black lines), indicating one-dimensional data.

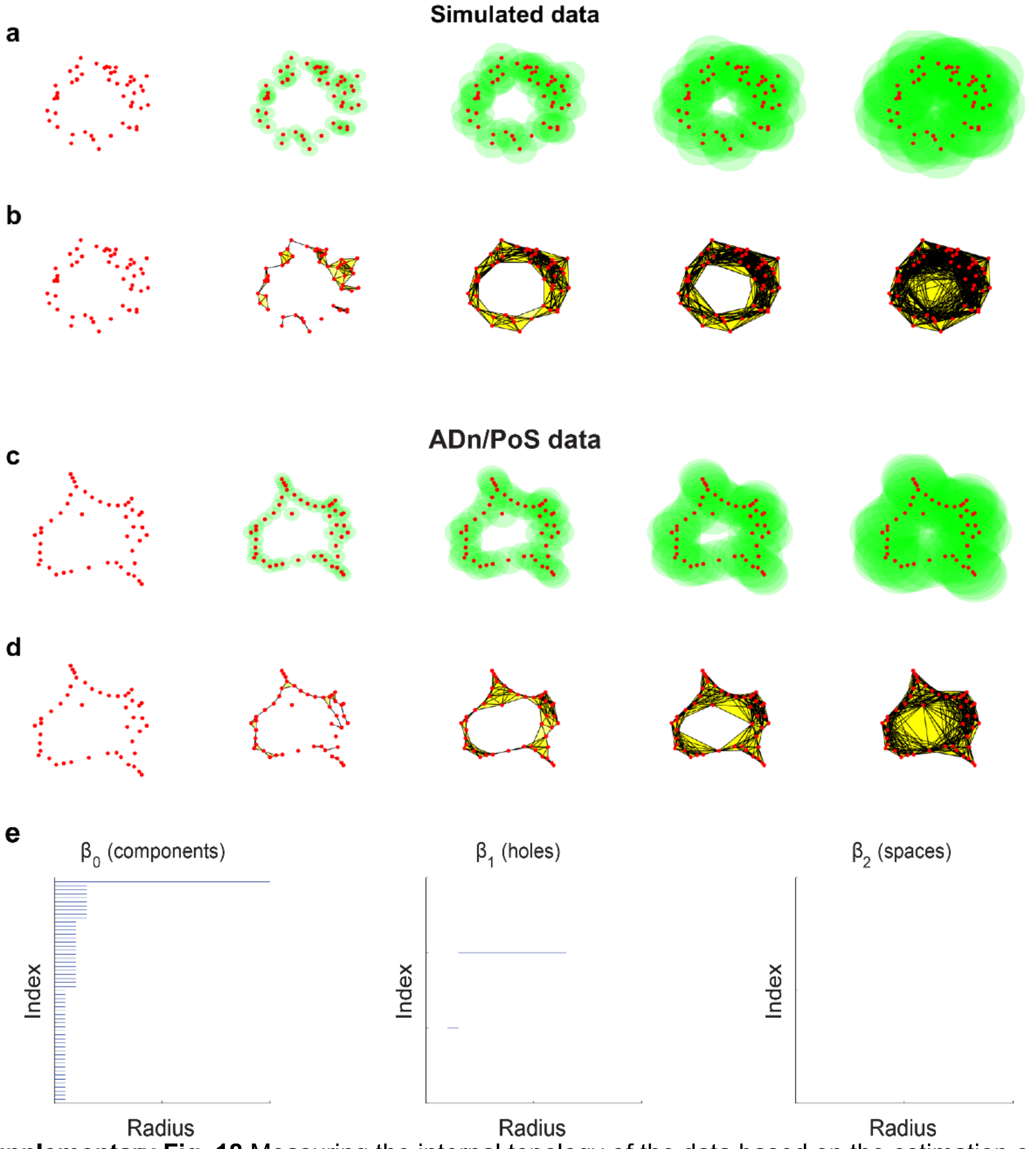

Supplementary Fig. 18 Measuring the internal topology of the data based on the estimation of the numbers of components, holes, and spaces. **a-b** Simulated data with a ring topology. **a** Simulated data points (red dots) and an increasing radius (from left to right), represented by filled green circles around each data point. **b** The connections (black lines) between data points with overlapping surrounding spheres for different radii of the sphere (increasing from left to right). Triplets (cliques of three data points that are all interconnected) are marked by yellow areas. **c-d** Head direction data. **c** Representative data points (centers of mass of clustered data, red dots) and an increasing radius (from left to right), represented by filled green circles around each data point. **d** The connections (black lines) between data points with overlapping surrounding spheres for different radii of the sphere (increasing from left to right). Triplets (cliques of three data points that are all interconnected) are marked by yellow areas. **e** Quantifying the internal topology of the ADn/PoS data by estimating the number of separate components (β_0), number of holes (β_1), and number of spaces (β_2). We observed one component, one hole, and zero spaces for a wide range of radii, as expected for a ring topology.

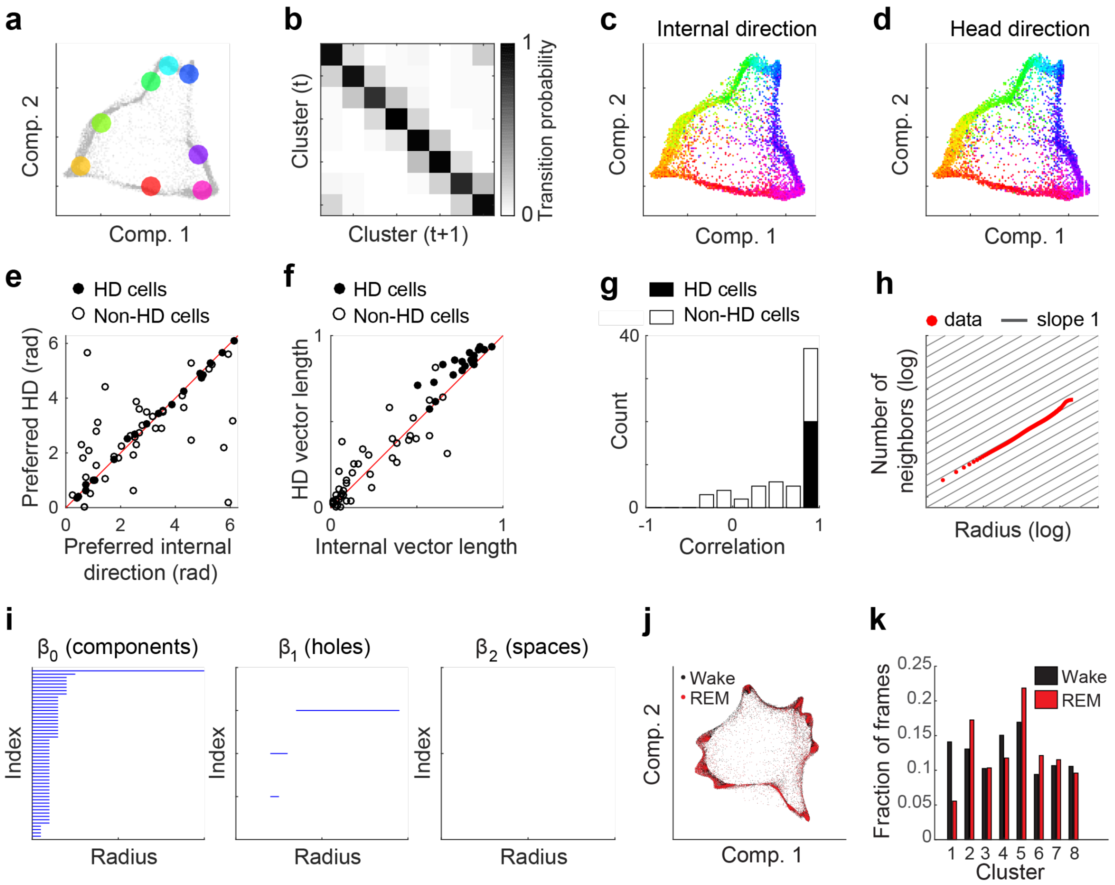

Supplementary Fig. 19 Internal representation of head direction during REM sleep. **a** Clustering of the data in Fig. 4C. The centers of mass of the clusters (colored dots) are overlaid on the data points in Fig. 4C (shaded gray). **b** The transition matrix (i.e., the probability of a data point to be in cluster *i* given that the preceding data point was in cluster *j*) shows that consecutive data points are more likely to be in the same or adjacent clusters. **c-d** The distribution of data points in the reduced dimensional space of neuronal activity, color coded according to the reconstructed internal direction **c** and according to the 'virtual' head direction **d**. The virtual head direction was obtained using a maximum likelihood decoder that was trained on data from wake periods. **e-f** The preferred direction **e** and Rayleigh vector length **f** calculated for the internal tuning curves during REM sleep (x-axis) and for the external tuning curves during wake periods (y-axis). Filled circles indicate significantly tuned head direction cells. **g** Distribution of correlation between internal (during REM) and external (during wake) tuning curves. Significantly tuned head direction cells are shown in black. **h** Estimation of internal dimension: Cumulative number of neighboring data points as a function of the radius in the reduced dimensionality space, plotted on a log-log scale. The slope of the data (red) is approximately one (black), indicating one-dimensional data. **i** Measuring the internal topology of the data based on the estimation of the numbers of components (β_0), holes (β_1), and spaces (β_2) reveals one component, one hole, and zero spaces for a wide range of radii. Overall, these results indicate that the relationships between the neuronal activity patterns are preserved even in the absence of sensory input, reflecting inherent computational properties of these circuits. **j** The data points in the reduced dimensional space of neuronal activity during wake periods and REM sleep are similarly distributed within the same ring structure. **k** Distribution of the data points in **j** across eight clusters.

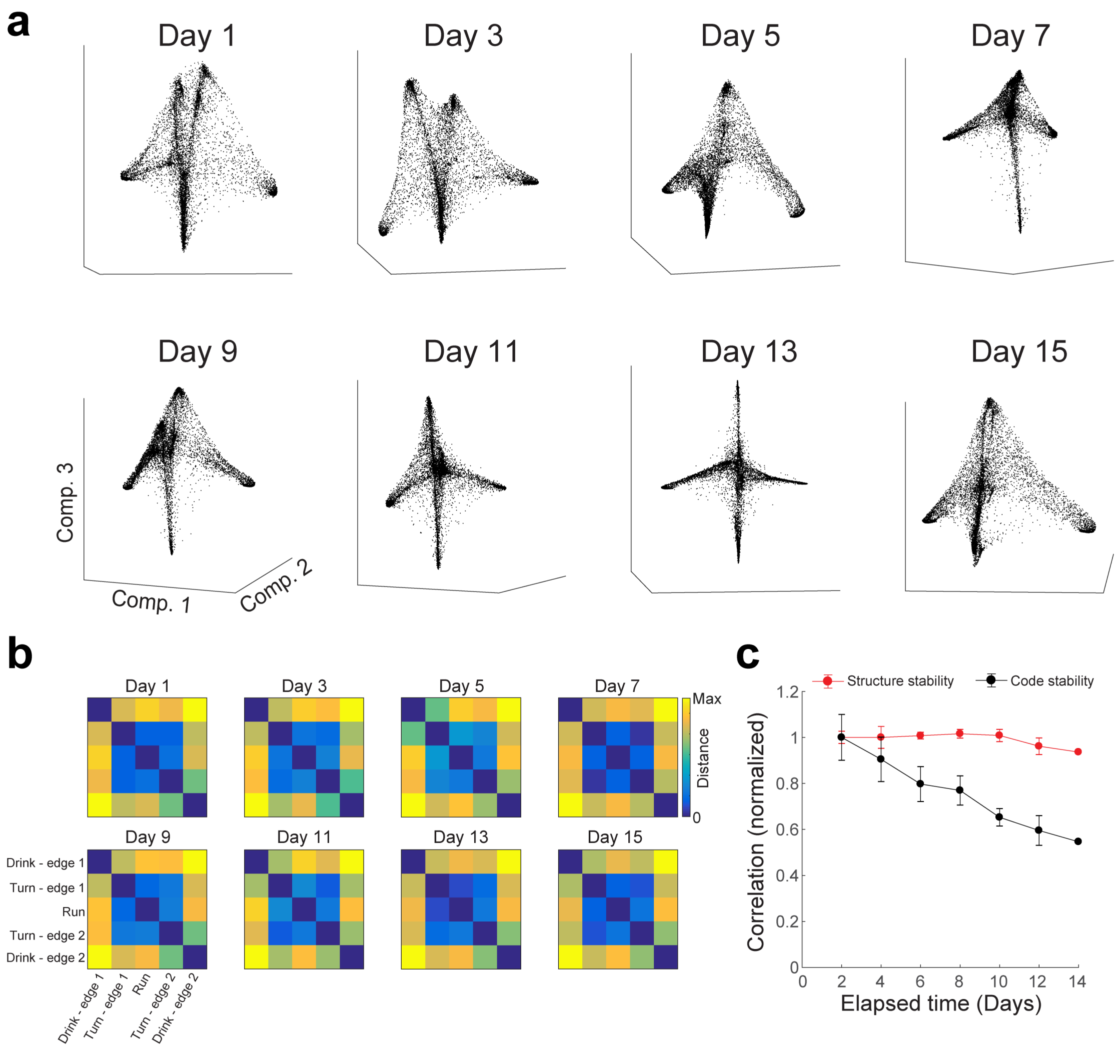

Supplementary Fig. 20 The internal structure of neuronal activity is maintained across days. **a** The distribution of data points in the first three dimensions of the reduced dimensional space of neuronal activity for hippocampal data from eight different days of the experiment. The data presented here is the same as in Fig. 5a. **b** Distances between the centers of mass of the different behavioral states (drinking in edge 1, turning in edge 1, running, drinking in edge 2, turning in edge 2) in the reduced dimensional space of neuronal activity are maintained across all days. For each pair of days, the correlation between the distance matrices was highest among all possible permutations (up to reflection, $p<0.02$ for all pairs of days). **c** Correlation (mean ± SD) between the distances matrix (red, structure stability) and the correlation between the average activity vectors (black, code stability) as a function of elapsed time. Both correlations were normalized to the value at the minimal elapsed time (2 days). While the population code gradually changes over time (slope of linear fit = -0.076, 95% confidence interval [-0.087 -0.065]), the structure is stable (slope of linear fit = -0.009, 95% confidence interval [-0.021 0.002]).
