# Supplementary material for: Revealing neural correlates of behavior without behavioral measurements
Source: Nat Commun. 2019 Oct 18;10:4745. doi: 10.1038/s41467-019-12724-2 (PMC6802184; doi:10.1038/s41467-019-12724-2)
Supplement: Supplementary file 3 — Description of Additional Supplementary Files [file 41467_2019_12724_MOESM3_ESM.pdf]

Supplementary Movie Legends

Supplementary Movie 1: Neuronal activity in the hippocampal CA1 reveals a high density of data points within a small number of clusters. The distribution of data points in the reduced dimensional space of neuronal activity from different view angles is shown.

Supplementary Movie 2: Different states of neuronal activity in the hippocampal CA1 correspond to different behavioral states, depending on the side of the linear track in which they occurred. Concatenated epochs of mouse behavior, which correspond to a given neuronal activity state are shown, and the neuronal activity state and index of the epoch are specified.

Supplementary Movie 3: Different states of neuronal activity in the ACC correspond to different behavioral states regardless of the side of the linear track. Concatenated epochs of mouse behavior correspond to a given neuronal activity state. The neuronal activity state and index of the epoch are specified.

Supplementary Movie 4: Neuronal activity in the ADn and PoS forms a ring topology in a reduced dimensional space with a non-periodic continuous trajectory. Top left: the trajectory of neuronal activity (moving dot) overlaid on the distribution of data points in the reduced dimensional space of neuronal activity. Bottom right: the actual head direction of the mouse, represented by the angle of the clock hand. Data points colored according to head direction.

Supplementary Movie 5: Neuronal activity in the ADn and PoS forms a ring topology in a reduced dimensional space during periods of REM sleep. The trajectory of neuronal activity (moving dot) overlaid on the distribution of data points in the reduced dimensional space of neuronal activity. The internal structure and the trajectory within it are maintained during REM sleep, when the head direction is mostly constant. Color represents the head direction as reconstructed by a maximum likelihood decoder that was trained on data from wake periods.

Supplementary Movie 6: Across-mice decoder infers the behavioral state and position of the animal, based on the mapping between the behavior and activity patterns in the hippocampus of another animal. Top left: The trajectory of neuronal activity (moving dot) for mouse 1 overlaid on the distribution of data points in the dimensionality reduced space of neuronal activity in mouse 1 (first two components). Top left: the corresponding trajectory of neuronal activity (moving dot) for mouse 1 overlaid on the distribution of data points in the dimensionality reduced space of neuronal activity in mouse 2 (first two components). Bottom left: Behavioral data from mouse 1. Bottom right: Reconstructed behavioral data of mouse 1 based on the mapping between the behavior and activity patterns.
